# Loss of SETD2-mediated H3K36me3 Drives CD8^+^ T Cell Exhaustion in Lung Cancer through CXCL-dependent PMN-MDSC Recruitment and Arginine Deprivation

**DOI:** 10.34133/research.1370

**Published:** 2026-08-05

**Authors:** Xuyu Gu, Junjie Zhu, Tao Ge, Keyi Chen, Yang Yang, Kaiqi Jin, Xinnan Xu

**Affiliations:** ^1^ Department of Oncology, Shanghai Pulmonary Hospital, School of medicine, Tongji University, Shanghai 200433, China.; ^2^ Department of Thoracic Surgery, Shanghai Pulmonary Hospital, School of medicine, Tongji University, Shanghai 200433, China.; ^3^ Innovation and Incubation Center (IIC), Shanghai Pulmonary Hospital, School of Medicine, Tongji University, Shanghai 200433, China.; ^4^ School of Materials Science and Engineering, Tongji University, Shanghai 201804, China.

## Abstract

Immune evasion in lung cancer is tightly regulated by epigenetic mechanisms. This study identifies key epigenetic drivers of immune escape in lung cancer and elucidates how they remodel the tumor microenvironment to induce CD8^+^ T cell exhaustion. We performed a CRISPR-negative screen targeting 159 epigenetic regulators (mEpi driver library) in a mouse model of lung metastasis and used single-cell sequencing, cell-based experiments, and chromatin analyses to identify mechanisms of immune escape. SET domain-containing protein 2 (SETD2) emerged as a critical suppressor of immune evasion. Loss of SETD2 markedly increased lung colonization and metastasis in mice with intact T cells, whereas this effect disappeared after T cell depletion. Mechanistically, SETD2 recruited adenine–thymine-rich interaction domain-containing protein 2 (ARID2) to maintain a repressive chromatin state at genes encoding the chemokines C-X-C motif chemokine ligand 1, 2, and 5. Loss of the SETD2–ARID2 axis increased secretion of these chemokines and recruited polymorphonuclear myeloid-derived suppressor cells, which inhibit T cell responses. These cells increased expression of free fatty acid receptor 2 (FFAR2), depleted arginine from the tumor environment, and impaired signaling required for T cell activation, driving cytotoxic T cells into terminal exhaustion. Depleting polymorphonuclear myeloid-derived suppressor cells or conditionally deleting FFAR2 in these cells restored T cell function and reduced metastasis of SETD2-deficient tumors. These findings show that loss of SETD2 creates an arginine-depleted, immunosuppressive tumor environment through a chemokine–FFAR2 pathway, revealing an epigenetic–metabolic immune checkpoint that may inform combination treatments for lung cancer.

## Introduction

Worldwide, lung cancer is still the deadliest form of cancer, claiming about 1.8 million lives every year while around 2 million new cases are diagnosed [1]. Historically, treatment options included surgery, platinum-based chemotherapy, and radiation therapy [2]. However, the introduction of immune checkpoint inhibitors, particularly targeting the programmed cell death protein 1 (PD-1)/programmed cell death ligand 1 (PD-L1) pathway, has substantially altered treatment protocols [3,4]. Despite these advancements, many patients develop either primary or acquired resistance, often due to a “cold” tumor microenvironment (TME) that lacks adequate T cell infiltration or activation [5,6]. To achieve stronger treatment outcomes and increase the proportion of patients who gain real advantages from immunotherapy, it is critically important to deeply understand the strategies tumors use to escape recognition and attack by the immune system.

The TME is a dynamic and multifaceted environment composed of cancer cells, stromal fibroblasts, and a variety of immune cells, such as tumor-associated macrophages, cytotoxic T lymphocytes, and myeloid-derived suppressor cells (MDSCs) [7]. Tumor cells in lung cancer often adopt intricate methods to alter this environment. By secreting immunosuppressive cytokines or increasing the expression of inhibitory ligands, cancer cells can effectively shield themselves from immune recognition [8]. The shift from an immune-active “hot” tumor to an immune-excluded “cold” tumor is often governed by both the genetic and epigenetic characteristics of the tumor cells themselves [9].

Epigenetic modifications, such as DNA methylation patterns and various histone modifications, have been identified as critical drivers of lung cancer progression. These epigenetic changes function as “master switches”, shutting down protective tumor suppressor genes or boosting harmful oncogenic pathways, all while leaving the underlying genetic sequence unchanged [10]. In cancer, such epigenetic disruptions can silence tumor-associated antigens, hinder antigen presentation, impair immune cell movement, and foster an immunosuppressive TME [11], all of which contribute to immune evasion. Epigenetic modifications stand apart from genetic mutations in one critical respect: They are reversible in nature, making the enzymes responsible for these changes promising targets for therapeutic strategies [12]. A deeper understanding of how these epigenetic modifications influence the tumor–immune interaction is essential for developing future combination therapies.

A prominent player in this epigenetic landscape is SET domain-containing 2 (SETD2), the principal mammalian methyltransferase responsible for canonical trimethylation of histone H3 at lysine-36 (H3K36me3) [13,14]. This modification is linked to several processes including transcription elongation, RNA splicing, DNA repair, and chromatin organization [15–17]. SETD2 mutations or deletions often disrupt the epigenetic landscape by depleting H3K36me3, which occurs in multiple cancer types, including clear cell renal cell carcinoma [18], prostate cancer [13], hematological cancers [19], and colorectal cancer [20]. Among the 10,953 patients analyzed in The Cancer Genome Atlas, 521 (4.76%) had SETD2 mutations [21]. SETD2 mutations occur in approximately 9% of lung adenocarcinomas, and SETD2 loss has been shown to promote Kras^G12D^-driven lung tumorigenesis [22]. While SETD2’s role in maintaining DNA integrity is well established [23], its impact on the immune landscape remains an emerging area of research.

The motivation for this study stems from the need to connect SETD2-mediated epigenetic silencing with the metabolic exhaustion of T cells. Specifically, this research explores how the loss of the SETD2–H3K36me3 axis results in the release of proinflammatory chemokines (C-X-C motif chemokine ligand 1/2/5 [CXCL1/2/5]), which, in turn, attract polymorphonuclear MDSCs (PMN-MDSCs) to the TME. By studying how SETD2 maintains a “closed” chromatin state at chemokine loci, this study aims to uncover a novel epigenetic–metabolic checkpoint. Understanding this pathway sheds light on how a single epigenetic change can transform the lung TME into an arginine-depleted environment, pushing CD8^+^ T cells into terminal exhaustion and promoting metastatic progression.

## Results

### SETD2 mutation promotes escape of distant lung cancer cells from T cell immunity and augments in vivo tumor growth

Murine Lewis lung carcinoma 3LL cells were implanted into C57BL/6 mice or nude mice via the tail vein at different cell doses (Fig. S1A). The results showed that fewer than 4 million (4M) 3LL cells failed to colonize lung tissue in C57BL/6 mice, whereas injection of only 2M 3LL cells was sufficient to form metastatic nodules in the lungs of nude mice (Fig. S1B and C). Given that C57BL/6 mice possess functional T cells, CD4^+^ or CD8^+^ T cells were depleted using neutralizing antibodies (Fig. S1D). Flow cytometry analysis confirmed a marked reduction of CD4^+^ or CD8^+^ T cells in both lung tissue and peripheral blood in these mice following antibody administration (Fig. S1E and F). Following T cell depletion, injection of 2M 3LL cells also led to the formation of lung metastatic nodules in C57BL/6 mice (Fig. S1G). These results indicated that T-cell-mediated immunity restricted the colonization of circulating lung cancer cells in tissues.

**Fig. 1.**
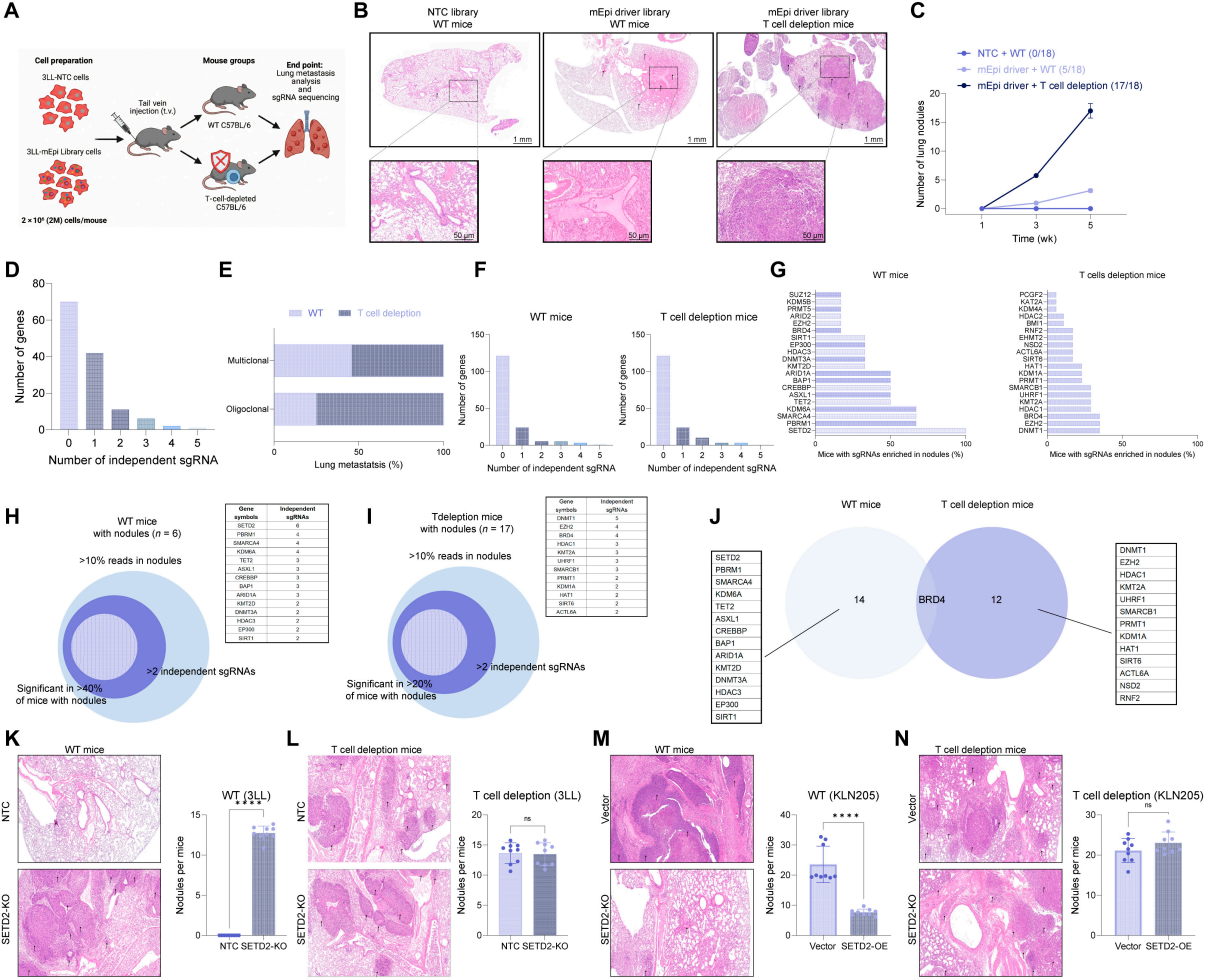
Identification of key epigenetic drivers of immune evasion in lung cancer through in vivo functional genomic screening. (A) Experimental workflow illustrating tail vein injection of mEpi driver single-guide RNA (sgRNA) library-infected 3LL cells into immunocompetent wild-type (WT) mice or antibody-mediated T-cell-depleted mice. (B) Representative histological images of lung tissues from each group. (C) Quantification and comparison of lung surface metastatic nodules across groups. (D) Amplicon sequencing of DNA extracted from metastatic lesions. (E) Analysis of monoclonal versus oligoclonal origins of metastatic lesions. (F) Schematic illustration of sgRNA enrichment-based screening thresholds. (G) Distribution analysis of sgRNA occurrence frequencies in WT versus T-cell-depleted mice. (H to J) Top candidate genes significantly enriched in WT (immune-intact) mice and T-cell-depleted mice. (K) Quantification of lung metastatic nodules formed by nontargeting control (NTC) or SET domain-containing protein 2 (SETD2)-knockout (KO) 3LL cells in WT mice. (L) Quantification of lung metastatic nodules formed by NTC or SETD2-KO 3LL cells in T-cell-depleted mice. (M) Quantification of lung metastatic nodules formed by control or SETD2-overexpressing KLN205 cells in WT mice. (N) Quantification of lung metastatic nodules formed by control or SETD2-overexpressing KLN205 cells in T-cell-depleted mice. Data are presented as bars and dots, with each dot representing data from one independent experiment. ****P < 0.0001. ns, nonsignificant; t.v., tail vein.

In addition, compared with normal lung tissue from C57BL/6 mice, metastatic lesions formed by 3LL cells exhibited a significant increase in infiltrating CD4^+^ and CD8^+^ T cells (Fig. S1H). The proportions of regulatory T cells (Tregs; Foxp3^+^CD4^+^) and exhausted CD8^+^ T cells (PD-1^+^TIM3^+^CD8^+^) were also markedly elevated in metastatic nodules (Fig. S1I). Our previous review has demonstrated the critical contribution of epigenetic modifications to the survival of lung adenocarcinoma cells under immune pressure [24]. Given these insights, a single-guide RNA (sgRNA) library targeting epigenetic regulatory enzymes (mEpi driver library), including chromatin remodelers, histone modification enzymes, and DNA methylation enzymes, was constructed. The library contained 1,684 sgRNAs targeting 159 genes, with an average of 4 sgRNAs per gene, together with 100 nontargeting controls (NTCs), and was used to infect 3LL cells. The cumulative distribution of sgRNA read counts in library-infected 3LL cells before injection indicated that more than 95% of sgRNAs were covered by over 100 reads (Fig. S1J and K). Subsequently, 3LL cells (2M) infected with the mEpi driver library or NTCs were implanted into C57BL/6 mice via the tail vein (Fig. 1A). Metastatic nodules were observed in the lungs of one-third of the mice receiving library-infected cells, whereas no metastatic lesions were detected in mice receiving NTC-infected cells (Fig. 1B and C).

To investigate clonal origins, all 50 metastatic lesions identified in wild-type (WT; immune-intact) mice and a randomly selected subset of 50 lesions from T-cell-depleted mice were microdissected individually. Deep amplicon sequencing of these isolated lesions uncovered a pronounced impact of adaptive T cell immunity on the monoclonal versus oligoclonal architecture of metastases (Fig. 1D and E).

To further identify critical genes involved in metastasis and immune evasion in tumor cells, the following screening criteria were applied: (a) enrichment of more than 2 independent sgRNAs per gene in metastatic lesions (Fig. 1F) and (b) an occurrence frequency greater than 40% in WT mice and greater than 20% in T-cell-deficient mice (Fig. 1G). Using these criteria, 14 genes were identified in WT (immune-intact) mice, and 13 genes were identified in T-cell-depleted mice (Fig. 1H to J). Among these, SETD2 was the gene specifically enriched in WT mice, indicating its potential involvement in immune regulation.

Correlation analysis using The Cancer Genome Atlas datasets showed that WT-specific genes exhibited strong positive correlations with immune-related genes, including CD8 α chain (CD8A), granzyme B (GZMB), granzyme A (GZMA), and perforin (PRF1) (Fig. S2A to D).

SETD2 expression was found to be significantly higher in 3LL cells than in KLN205 cells (Fig. S2E). Three sgRNAs were designed to generate SETD2-knockout (KO) 3LL cell lines (Fig. S2F). Western blot (WB) analysis of SETD2 protein levels and H3K36me3 modification confirmed KO efficiency (Fig. S2G). In parallel, SETD2 was overexpressed in KLN205 cells (Fig. S2H). Both 3LL and KLN205 cells were then injected into WT or T-cell-depleted mice via the tail vein (Fig. S2I).

SETD2-deficient 3LL cells formed significantly more lung metastatic lesions in WT mice compared with NTC controls, whereas no significant difference was identified in T-cell-depleted mice (Fig. 1K and L). Conversely, SETD2-overexpressing (OE) KLN205 cells formed significantly fewer lung metastatic lesions in WT mice, with no significant difference observed in T-cell-depleted mice (Fig. 1M and N). To assess whether SETD2 loss directly affects tumor-cell growth, we compared the in vitro proliferation of NTC and SETD2-KO 3LL cells. Cell counting kit-8 assays showed no significant difference in proliferation under standard culture conditions (Fig. S2J). Together with the observation that SETD2-KO cells did not show a metastatic advantage in T-cell-depleted mice, these data suggest that the enhanced metastatic growth of SETD2-deficient tumors is largely dependent on immune-mediated mechanisms rather than increased cell-autonomous proliferation.

### Loss of SETD2 reshapes the TME and impairs T cell function

To examine the correlation between SETD2 and tumor immune response, SETD2 expression was analyzed across multiple datasets. In 2 cohorts of patients who received anti-PD-1 therapy, SETD2 expression was considerably lower in nonresponders than in responders (Fig. S3A and B). Moreover, based on Response Evaluation Criteria in Solid Tumors criteria, SETD2 expression progressively increased in patients classified as having stable disease, partial response, or complete response following anti-PD-1 therapy (Fig. S3C). In addition, SETD2 expression showed a significant positive correlation with cytotoxic T lymphocyte infiltration (Fig. S3D to F).

As shown in Fig. S1I, metastatic nodules formed by 3LL cells exhibited markedly higher numbers of exhausted T cells and Tregs than normal lung tissue. Therefore, these populations were further examined in metastatic lesions formed by injection of 2M 3LL cells. The results showed that metastatic lesions derived from SETD2-KO 3LL cells contained significantly increased proportions of exhausted T cells and Tregs (Fig. S4A and B). Conversely, metastatic nodules formed by SETD2-OE KLN205 cells displayed markedly reduced numbers of exhausted T cells and Tregs (Fig. S4C and D).

**Fig. 2.**
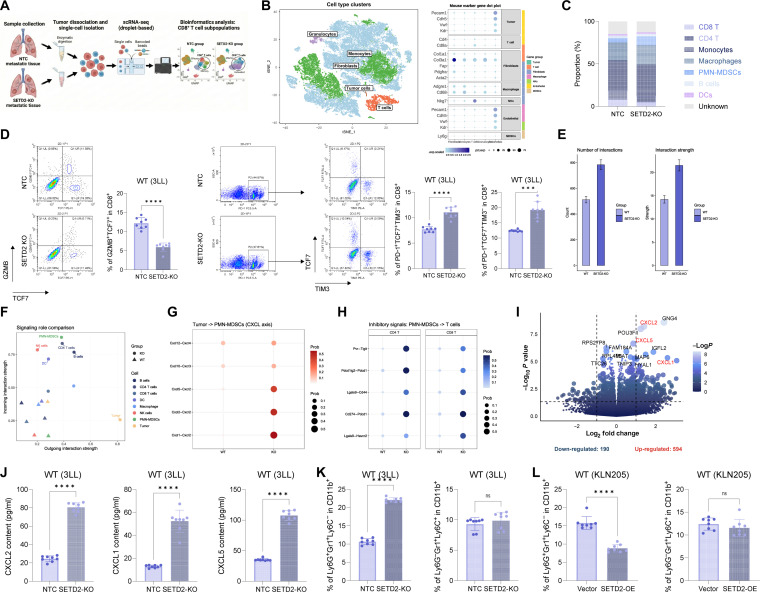
SET domain-containing protein 2 (SETD2) loss induces tumor microenvironment (TME) remodeling and altered CD8^+^ T cell functional states in vivo. (A) Single-cell RNA sequencing (scRNA-seq) analysis of CD8^+^ T cell subsets in metastatic lesions derived from nontargeting control (NTC) and SETD2-knockout (KO) tumors. (B) t-distributed stochastic neighbor embedding (tSNE) visualization of CD8^+^ T cell subset clustering. NKs, natural killer cells. (C) Quantification of immune cell subset abundance in each group. DCs, dendritic cells. (D) Flow cytometry analysis of effector (GZMB^+^TCF7^+^), progenitor exhausted (PD-1^+^TCF7^+^), and terminally exhausted (PD-1^+^TIM3^+^) CD8^+^ T cell populations in NTC or SETD2-KO 3LL cell-derived metastatic nodules. PE, phycoerythrin; SSC, side scatter. (E) CellChat-based analysis of global intercellular communication number and strength in NTC and SETD2-KO tumors. (F) CellChat centrality analysis evaluating outgoing signaling strength of tumor cells and signal reception by immune cell subsets. (G) CellChat analysis of communication probabilities for the tumor-derived C-X-C motif chemokine ligand (CXCL)–CXCR2 axis. (H) Analysis of inhibitory signaling axes between polymorphonuclear myeloid-derived suppressor cells (PMN-MDSCs) and T cells, including Galectin-9/TIM3 and PD-L1/PD-1. (I) Heatmap of differentially expressed genes from bulk RNA-seq analysis of NTC and SETD2-KO nodules. (J) Quantification of C-X-C motif chemokine ligand 1/2/5 (CXCL1/2/5) expression levels in metastatic lesions formed by NTC or SETD2-KO 3LL cells. (K) Flow cytometry quantification of PMN-MDSCs (CD45^+^Ly6G^+^Ly6C^−^Gr1^+^CD11b^+^) and monocytic MDSCs (MN-MDSCs) (CD45^+^Ly6G^−^Ly6C^+^Gr1^+^CD11b^+^) in metastatic lesions formed by NTC or SETD2-KO 3LL cells. (L) Flow cytometry quantification of PMN-MDSCs and MN-MDSCs in metastatic lesions formed by NTC or SETD2-OE KLN205 cells. Data are presented as bars and dots, with each dot representing data from one independent experiment. ***P < 0.001 and ****P < 0.0001. ns, nonsignificant.

To determine whether SETD2 directly affects T cell activity, an in vitro coculture system was established. Peripheral blood mononuclear cells were isolated from mice, stimulated with anti-CD3/CD28 antibodies, and purified CD8^+^ T cells were obtained using anti-CD8 antibodies. Following interleukin-2 (IL-2)-mediated activation, CD8^+^ T cells were cocultivated with 3LL cells at different ratios (effector-to-target = 1:1 or 1:2) (Fig. S4E). As expected, direct in vitro coculture with NTC 3LL cells established a baseline impairment of CD8^+^ T cell activation, characterized by reduced T cell proliferation (Fig. S4F), decreased proportions of GZMB^+^CD8^+^ T cells (Fig. S4G), and diminished cytotoxic activity against target cells (Fig. S4H). Notably, coculture with SETD2-KO 3LL cells did not further exacerbate this immunosuppression; T cell functional metrics remained entirely comparable to those in the NTC coculture group (Fig. S4F to H). This comparable in vitro suppression is a critical distinction, as it indicates that the profound terminal T cell exhaustion observed in SETD2-deficient tumors in vivo (Fig. 2D) is not driven by enhanced direct tumor-to-T-cell interactions. Rather, these findings strongly suggest that SETD2 loss drives immune evasion indirectly, necessitating the recruitment and reorganization of additional intermediate immunosuppressive cellular components within the TME.

To test this hypothesis, single-cell RNA sequencing (scRNA-seq) was performed on tumor nodules derived from NTC or SETD2-KO cells. A marked decrease in the overall abundance of CD8^+^ T cells was identified in SETD2-KO tumors (Fig. 2A to C). Further subclustering analysis revealed significant increases in progenitor exhausted CD8^+^ T cells (PD-1^+^TCF7^+^) and terminally exhausted CD8^+^ T cells (PD-1^+^TIM3^+^), along with a pronounced decrease in functional GZMB^+^TCF7^+^ CD8^+^ T cells (Fig. 2D).

To further elucidate mechanisms associated with CD8^+^ T cell exhaustion in the SETD2-deficient TME, we performed CellChat analysis to infer ligand–receptor-mediated communication networks from the scRNA-seq dataset. Compared with NTC tumors, SETD2-KO tumors exhibited a global reorganization of signaling networks, with significantly increased numbers and overall strengths of predicted cell–cell interactions (Fig. 2E). Network centrality analysis further indicated that SETD2-KO tumor cells showed enhanced outgoing signaling activity, with PMN-MDSCs representing one of the major recipient populations within the remodeled communication network (Fig. 2F). These findings suggested that SETD2 loss in tumor cells may alter the TME through paracrine signaling to immunosuppressive myeloid cells.

Among the enhanced tumor-cell-derived outgoing pathways, CellChat identified the CXCL pathway as the most pronounced. In particular, tumor-derived CXCL1, CXCL2, and CXCL5 were predicted to interact strongly with CXCR2 on PMN-MDSCs in SETD2-KO tumors (Fig. 2G). Because this axis showed strong tumor-to-PMN-MDSC directionality and direct relevance to granulocytic myeloid cell recruitment, it was prioritized for experimental validation.

CellChat also predicted increased inhibitory signaling from PMN-MDSCs to CD8^+^ T cells, including Galectin-9/TIM3 and PD-L1/PD-1 interactions (Fig. 2H). These analyses indicate that SETD2 loss may promote the recruitment of PMN-MDSCs via the CXCL1/2/5–CXCR2 axis, which, in turn, induces terminal T cell exhaustion within the local TME.

Consistent with these findings, bulk RNA-seq analysis comparing NTC and SETD2-KO tumors demonstrated significantly elevated expression of CXCL1, CXCL2, and CXCL5 in SETD2-KO nodules (Fig. 2I and J). In addition, flow cytometry analysis of CD45^+^Ly6G^+^Ly6C^−^Gr1^+^CD11b^+^ (PMN-MDSCs) and CD45^+^Ly6G^−^Ly6C^+^Gr1^+^CD11b^+^ (monocytic MDSCs [MN-MDSCs]) cell populations within nodules formed by 3LL or KLN205 cells showed that PMN-MDSCs were significantly increased in SETD2-KO tumors, whereas no significant change was observed in MN-MDSCs (Fig. 2K). Conversely, nodules formed by SETD2-OE cells exhibited a marked reduction in PMN-MDSCs, with MN-MDSC levels remaining unchanged (Fig. 2L).

### SETD2 modulates the TME through PMN-MDSC-mediated mechanisms

To determine whether PMN-MDSCs mediated the effects of SETD2 loss on the TME, mice injected with SETD2-KO cells were treated with anti-Ly6G antibodies to deplete Ly6G^+^ granulocytic myeloid cells (Fig. S5A). Following anti-Ly6G treatment, the number of lung nodules was substantially decreased (Fig. S5B and C). In parallel, the abundance of effector T cells within metastatic nodules was markedly increased (Fig. S5D), while the proportion of terminally exhausted T cells was considerably decreased (Fig. S5E). Because Ly6G is also expressed by neutrophil-lineage cells, we further sorted PMN-MDSC-like cells (CD45^+^Ly6G^+^Ly6C^−^Gr1^+^CD11b^+^) from tumors and had them cocultured with CD8^+^ T cells. Coculture with PMN-MDSC-like cells from anti-Ly6G-treated mice showed reduced suppression of CD8^+^ T proliferation and effector function (Fig. S5F and G).

**Fig. 3.**
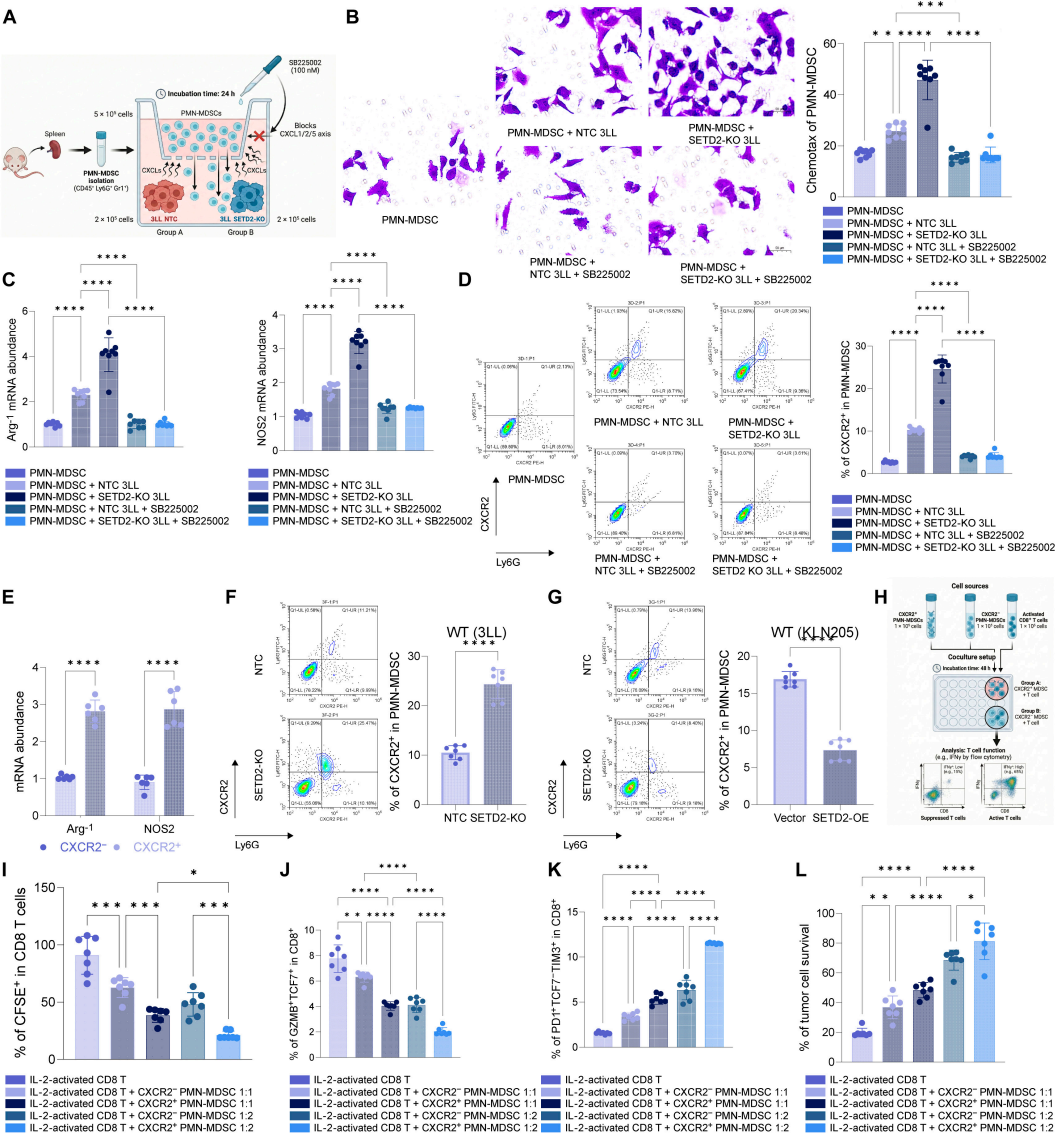
SET domain-containing protein 2 (SETD2) loss drives C-X-C motif chemokine ligand (CXCL)–CXCR2 axis-mediated recruitment and suppressive polarization of polymorphonuclear myeloid-derived suppressor cells (PMN-MDSCs). (A) Schematic of the Transwell coculture system of nontargeting control (NTC) or SETD2-knockout (KO) 3LL cells with PMN-MDSCs, followed by the treatment of CXCL1/2/5–CXCR2 inhibitor SB225002. (B) Quantification of PMN-MDSC chemotaxis toward tumor cells across groups. (C) Quantitative polymerase chain reaction (qPCR) analysis of arginase-1 (Arg1) and nitric oxide synthase 2 (NOS2) mRNA expression in PMN-MDSCs after coculture with tumor cells. (D) Flow cytometry analysis of the proportion of CXCR2^+^ PMN-MDSCs. (E) Flow cytometry assessment of Arg1 and NOS2 levels in CXCR2^+^ versus CXCR2^−^ PMN-MDSCs. (F) Flow cytometry quantification of CXCR2^+^ PMN-MDSCs in SETD2-KO metastatic nodules. (G) Flow cytometry quantification of CXCR2^+^ PMN-MDSCs in SETD2-overexpressing (OE) metastatic nodules. (H) Schematic of the functional coculture system of CXCR2^+^ or CXCR2^−^ PMN-MDSCs with CD8^+^ T cells. IFNγ, interferon-γ. (I) Carboxyfluorescein diacetate succinimidyl ester (CFSE) dilution assay measuring CD8^+^ T cell proliferation in coculture. (J and K) Flow cytometry analysis of effector (GZMB^+^TCF7^+^) (J) and terminally exhausted (PD-1^+^TIM3^+^) (K) CD8^+^ T cell subsets. (L) Lactate dehydrogenase (LDH) release assay quantifying CD8^+^ T-cell-mediated cytotoxicity against tumor cells. Data are presented as bars and dots, with each dot representing data from one independent experiment. *P < 0.05, **P < 0.01, ***P < 0.001, and ****P < 0.0001.

To further define the underlying mechanism, NTC or SETD2-KO 3LL cells were plated in the lower chamber of a Transwell system, while PMN-MDSCs (CD45^+^Ly6G^+^Ly6C^−^Gr1^+^CD11b^+^) from mouse spleens were loaded in the upper chamber, with or without the competitive CXCL1/2/5–CXCR2 inhibitor SB225002 (Fig. 3A). SETD2-KO cells exhibited a strengthened capacity to attract PMN-MDSCs compared with NTC 3LL cells, and treatment with SB225002 suppressed the PMN-MDSC chemotaxis toward 3LL cells (Fig. 3B). To test the in vivo requirement for CXCL1/2/5 signaling, mice bearing SETD2-KO tumors were treated with a neutralizing antibody cocktail against CXCL1, CXCL2, and CXCL5. Flow cytometry analysis showed that chemokine neutralization reduced the accumulation of CXCR2^+^ Ly6G^+^ granulocytic/PMN-MDSC-like cells compared with isotype-treated controls (Fig. S5H and I). This was accompanied by increased effector CD8^+^ T cells (GZMB^+^TCF7^+^) and reduced progenitor exhausted CD8^+^ T cells (PD-1^+^TCF7^+^) in the TME (Fig. S5J and K). These findings support a functional role for CXCL1/2/5 in promoting CXCR2^+^ PMN-MDSC recruitment and CD8^+^ T cell suppression in SETD2-deficient tumors.

Notably, coculture with NTC 3LL cells induced a substantial increase in Arg1 (arginase-1) and NOS2 (nitric oxide synthase 2) expression in PMN-MDSCs, with these trends strengthened upon SETD2 loss in 3LL cells (Fig. 3C). In addition, the proportion of CXCR2^+^ PMN-MDSCs was increased following 3LL and, in particular, SETD2-KO 3LL cell coculture (Fig. 3D). These increases were negated upon SB225002 administration. Moreover, CXCR2^+^ PMN-MDSCs expressed higher levels of Arg1 and NOS2 than their CXCR2^−^ counterparts (Fig. 3E). Consistent with these observations, a significant increase in CXCR2^+^ PMN-MDSCs was detected in SETD2-KO metastatic nodules (Fig. 3F), whereas this population was reduced in SETD2 overexpression-formed metastatic lesions (Fig. 3G).

To directly assess the influence of PMN-MDSCs on CD8^+^ T cell activity, CXCR2^+^ or CXCR2^−^ PMN-MDSCs were cocultivated with CD8^+^ T cells (Fig. 3H). CXCR2^+^ PMN-MDSCs exerted a significantly stronger suppressive effect on CD8^+^ T cell proliferation, effector differentiation, and cytotoxic function than CXCR2^−^ PMN-MDSCs (Fig. 3I to L).

### CXCL1/2/5–CXCR2 signaling activates STAT3 and drives excessive l-arginine uptake, resulting in impaired T cell receptor signaling

Arg1 and NOS2 competitively utilize the shared metabolic substrate l-arginine: Arg1 converts it into urea and ornithine, while NOS2 converts it into nitric oxide. This competition for l-arginine leads to a pronounced arginine deficit within the TME [25]. Because CD8^+^ T cell activation, protein synthesis, and stability of the CD3ζ chain are highly dependent on extracellular arginine availability, such metabolic deprivation may directly induce cell cycle arrest in T cells [26]. These insights suggest that recruitment of PMN-MDSCs by SETD2 loss through chemokine signaling may represent only the initial step; more critically, SETD2 deficiency induces metabolic reprogramming within MDSCs, enforcing an arginine-depletion-mediated “metabolic checkpoint” that irreversibly drives CD8^+^ T cells from an effector state toward terminal exhaustion through epigenetic remodeling.

Consistent with this hypothesis, CXCR2^+^ PMN-MDSCs exhibited significantly higher l-arginine uptake compared with CXCR2^−^ PMN-MDSCs (Fig. S6A). In coculture assays, CD8^+^ T cells cultured with CXCR2^+^ PMN-MDSCs showed a marked reduction in CD3ζ chain expression, accompanied by decreased phosphorylation of ZAP70 (zeta-chain-associated protein kinase 70) and LCK (lymphocyte-specific protein tyrosine kinase) (Fig. S6B and C), as well as diminished Ca^2+^ influx (Fig. S6D). Notably, supplementation of exogenous l-arginine in the CXCR2^+^ PMN-MDSC-CD8^+^ T cell coculture system (Fig. S6E) partly negated the suppressive effects of CXCR2^+^ PMN-MDSCs on CD8^+^ T cell function (Fig. S6F to H). Having shown that tumor-intrinsic SETD2 loss promotes the metabolic activation of PMN-MDSCs, we next examined whether increased SETD2 expression in tumor cells could conversely suppress this process. To this end, we generated SETD2-OE 3LL cells and evaluated their ability to modulate primary PMN-MDSCs in vitro. Compared with PMN-MDSCs cocultured with control tumor cells, PMN-MDSCs exposed to SETD2-OE tumor cells exhibited reduced expression of the immunosuppressive metabolic enzymes ARG1 and NOS2, as well as the arginine-uptake-associated transporter free fatty acid receptor 2 (FFAR2) (Fig. S6I and J). These findings further support a bidirectional relationship between tumor cell SETD2 status and the establishment of an arginine-consumptive PMN-MDSC phenotype in the TME.

**Fig. 4.**
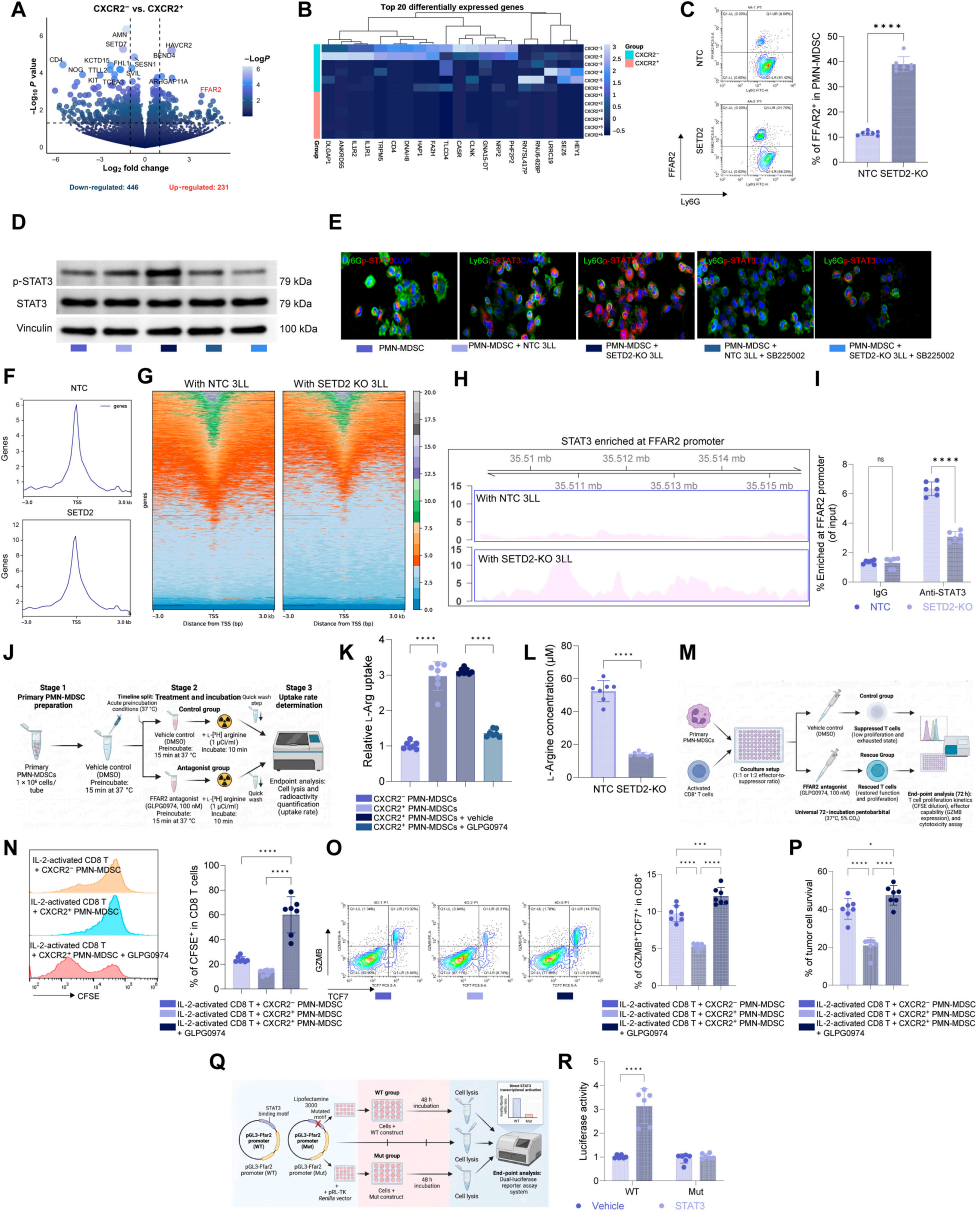
The signal transducers and activators of transcription 3 (STAT3)–free fatty acid receptor 2 (FFAR2) axis regulates arginine uptake and metabolic competition in polymorphonuclear myeloid-derived suppressor cells (PMN-MDSCs). (A) Heatmap of differentially expressed genes from bulk RNA-seq comparing CXCR2^+^ and CXCR2^−^ PMN-MDSCs. (B) Gene set enrichment analysis of Hallmark pathways showing normalized enrichment scores. (C) Flow cytometry quantification of FFAR2^+^ PMN-MDSCs in metastatic nodules formed by nontargeting control (NTC) or SET domain-containing protein 2 (SETD2)-knockout (KO) 3LL cells. (D) Western blot (WB) analysis of FFAR2 expression and STAT3 phosphorylation at Y705 and S727 in PMN-MDSCs across groups. (E) Immunofluorescence analysis quantifying nuclear translocation and localization of phosphorylated STAT3 in PMN-MDSCs across groups. (F) STAT3 chromatin immunoprecipitation sequencing (ChIP-seq) analysis showing average binding profiles across promoter-like genomic regions in PMN-MDSCs cocultured with NTC or SETD2-KO 3LL cells. (G) Genome-wide heatmap of STAT3 ChIP-seq signal enrichment. (H) Genome browser tracks showing STAT3 occupancy at the FFAR2 promoter locus. (I) ChIP-quantitative polymerase chain reaction (qPCR) validation of STAT3 enrichment at the FFAR2 promoter in CXCR2^+^ PMN-MDSCs. (J) Schematic of the l-[^3^H] arginine uptake assay in CXCR2^+^ PMN-MDSCs treated with vehicle or the FFAR2 antagonist GLPG0974. (K) Quantification of l-[^3^H] arginine uptake in CXCR2^+^ PMN-MDSCs after acute GLPG0974 treatment. (L) Quantification of extracellular l-arginine levels in tumor interstitial fluid from lung metastatic nodules formed by NTC or SETD2-KO 3LL cells. (M) Schematic of the coculture assay used to assess the effect of FFAR2 inhibition on CXCR2^+^ PMN-MDSC-mediated suppression of activated CD8^+^ T cells. (N) CD8^+^ T cell proliferation after coculture with CXCR2^+^ PMN-MDSCs in the presence of vehicle or GLPG0974. (O) Flow cytometry quantification of GZMB^+^CD8^+^ T cells after coculture with CXCR2^+^ PMN-MDSCs treated with vehicle or GLPG0974. (P) Cytotoxic activity of CD8^+^ T cells against 3LL tumor cells after coculture with CXCR2^+^ PMN-MDSCs treated with vehicle or GLPG0974. (Q) Schematic of the dual-luciferase reporter assay using the wild-type (WT) Ffar2 promoter or a promoter construct with mutations in the predicted STAT3-binding motif. Mut, mutant. (R) Luciferase activity driven by WT or STAT3-motif-mutant Ffar2 promoter constructs following STAT3 activation. Data are presented as bars and dots, with each dot representing data from one independent experiment. *P < 0.05, ***P < 0.001, and ****P < 0.0001.

To further elucidate the molecular basis of enhanced arginine consumption, bulk RNA-seq was performed to compare differentially expressed genes between CXCR2^−^ and CXCR2^+^ PMN-MDSCs, with FFAR2 identified as one of the most significantly up-regulated genes in CXCR2^+^ PMN-MDSCs (Fig. 4A). Gene set enrichment analysis using the Hallmark gene sets demonstrated that the signal transducer and activator of transcription 3 (STAT3) signaling pathway exhibited the highest normalized enrichment score (Fig. 4B). These findings prompted the hypothesis that CXCL1/2/5–CXCR2 signaling activated STAT3 nuclear translocation, thereby promoting FFAR2 expression.

To determine whether this regulatory axis was conserved across lung cancer models, we validated key findings in KLN205 cells. SETD2 overexpression in KLN205 cells significantly reduced Cxcl1, Cxcl2, and Cxcl5 expression (Fig. S6K). In PMN-MDSC education assays, primary PMN-MDSC-like cells cocultured with SETD2-OE KLN205 cells showed reduced expression of Arg1, Nos2, and Ffar2 compared with those cocultured with control KLN205 cells (Fig. S6L and M). These results support the conclusion that SETD2-dependent regulation of chemokine expression and PMN-MDSC metabolic activation is not restricted to the 3LL model.

Consistent with this hypothesis, increased FFAR2^+^ MDSCs were detected in metastatic nodules derived from SETD2-KO 3LL tumors (Fig. 4C). In PMN-MDSCs cocultured with 3LL cells, SETD2-KO coculture conditions resulted in markedly elevated FFAR2 expression and enhanced STAT3 phosphorylation, effects negated by the SB225002 treatment (Fig. 4D and E). Treatment of PMN-MDSCs cocultured with SETD2-KO 3LL cells using the STAT3-specific inhibitor Stattic (Fig. S7A) significantly reduced l-arginine uptake (Fig. S7B), decreased FFAR2 expression (Fig. S7C), and attenuated Arg1 and NOS2 transcript levels (Fig. S7D), without altering the proportion of CXCR2^+^ PMN-MDSCs (Fig. S7E). Furthermore, the supplementation of Stattic to the PMN-MDSC–CD8^+^ T cocultures (Fig. S7F) led to a pronounced reduction in PMN-MDSC-mediated immunosuppression of CD8^+^ T cells (Fig. S7G to I).

Furthermore, chromatin immunoprecipitation sequencing (ChIP-seq) analysis using anti-STAT3 revealed a substantial increase in STAT3 binding to promoter-like regions across the genome in PMN-MDSCs cocultured with SETD2-KO 3LL cells (Fig. 4F and G). Notably, enhanced STAT3 occupancy was detected at the FFAR2 promoter locus in PMN-MDSCs cocultured with SETD2-KO 3LL cells (Fig. 4H). ChIP-quantitative polymerase chain reaction (qPCR) further confirmed increased STAT3 enrichment at the FFAR2 promoter in CXCR2^+^ PMN-MDSCs (Fig. 4I). In addition, treatment with the CXCR2 inhibitor SB225002 in PMN-MDSCs cocultured with SETD2-KO 3LL cells also markedly suppressed FFAR2 expression and STAT3 activation (Fig. 4D and E).

To examine whether FFAR2 activity contributes to enhanced l-arginine uptake in CXCR2^+^ PMN-MDSCs, we performed l-[^3^H] arginine uptake assays using the FFAR2 antagonist GLPG0974 (Fig. 4J). Acute treatment with GLPG0974 (100 nM, 15 min) markedly reduced l-arginine uptake in CXCR2^+^ PMN-MDSCs compared with vehicle controls (Fig. 4K). Because this short-term treatment was unlikely to reflect transcriptional remodeling, these data suggest that FFAR2 activity rapidly regulates l-arginine uptake in CXCR2^+^ PMN-MDSCs.

To determine whether increased l-arginine uptake was associated with reduced extracellular arginine availability in vivo, we extracted tumor interstitial fluid from lung metastatic nodules and measured l-arginine levels. Consistent with the in vitro uptake assays, SETD2-KO tumors showed lower extracellular l-arginine levels than NTC tumors (Fig. 4L), indicating an arginine-depleted metabolic environment in SETD2-deficient tumors.

We next tested whether pharmacological inhibition of FFAR2 could attenuate PMN-MDSC-like cell-mediated T cell suppression in vitro. CXCR2^+^ PMN-MDSC-like cells were cocultured with activated CD8^+^ T cells in the presence of GLPG0974 (100 nM) (Fig. 4M). FFAR2 inhibition reduced the suppressive effect of CXCR2^+^ PMN-MDSC-like cells, as shown by increased CD8^+^ T cell proliferation (Fig. 4N), restored GZMB expression (Fig. 4O), and enhanced CD8^+^ T cell cytotoxicity against 3LL tumor cells (Fig. 4P). To further assess whether STAT3 directly regulates Ffar2 transcription, we analyzed the STAT3 ChIP-seq peak within the Ffar2 promoter and identified a putative STAT3-binding motif. We then performed dual-luciferase reporter assays using either the WT Ffar2 promoter or a promoter construct carrying mutations in this motif. STAT3 activation increased WT Ffar2 promoter activity, whereas mutation of the STAT3-binding motif markedly reduced this induction (Fig. 4Q and R). These data support direct transcriptional regulation of Ffar2 by STAT3 in PMN-MDSC-like cells.

### Ly6G^+^ granulocytic/PMN-MDSC-like cell-specific FFAR2 deletion reduces in vivo metastasis of SETD2-deficient lung cancer cells

**Fig. 5.**
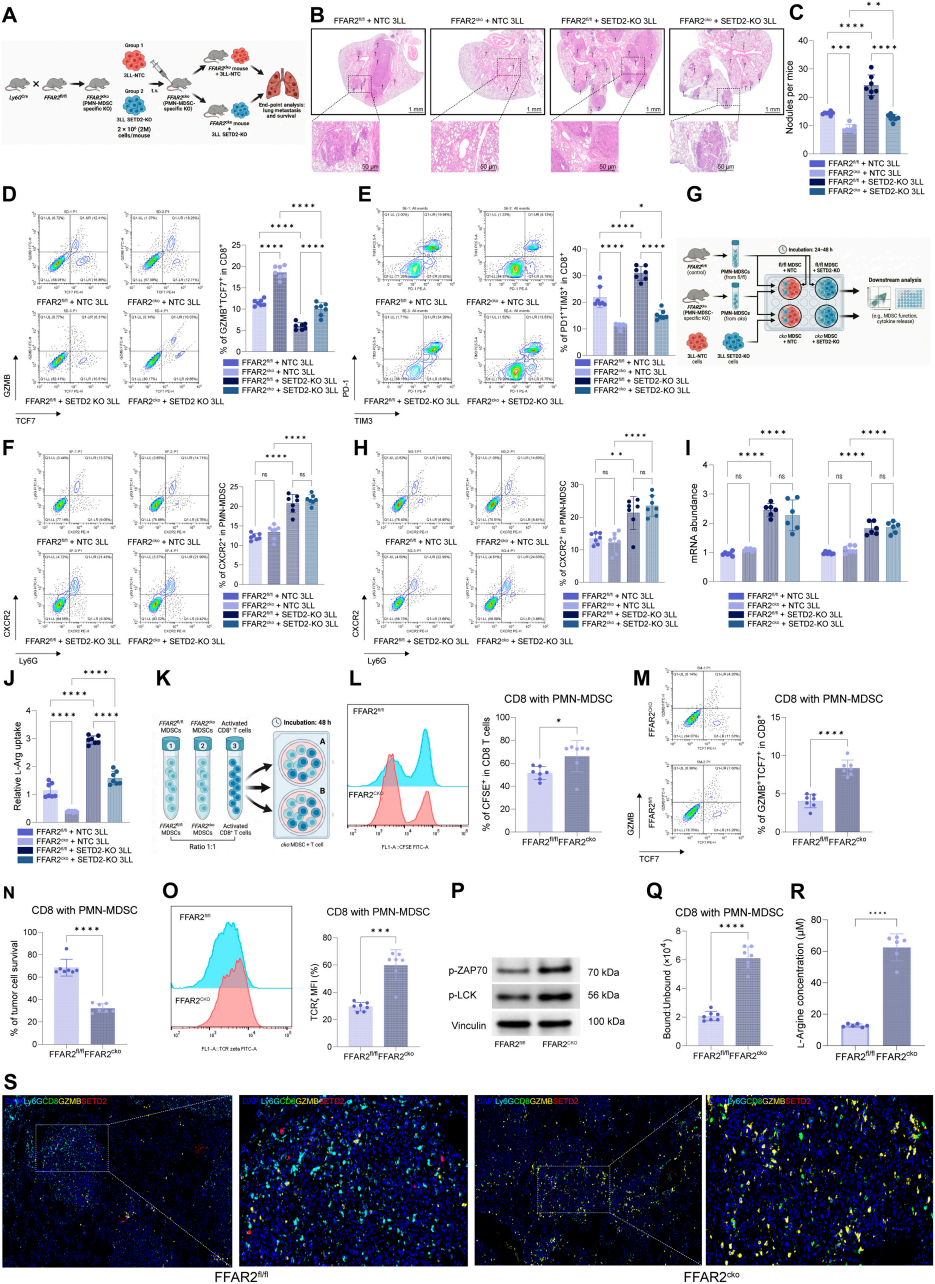
Ly6G^+^ granulocytic/polymorphonuclear myeloid-derived suppressor cell (PMN-MDSC)-like cell-specific FFAR2 deletion reduces in vivo metastasis of SET domain-containing protein 2 (SETD2)-deficient lung cancer cells. (A) Experimental workflow showing injection of nontargeting control (NTC) or SETD2-knockout (KO) 3LL cells into FFAR2^cko^ or FFAR2^fl/fl^ (control) mice. (B) Representative histological images of lungs from each group. (C) Quantification of lung surface metastatic nodules. (D) Flow cytometry analysis of GZMB^+^TCF7^+^ effector CD8^+^ T cells in metastatic nodules. (E) Flow cytometry analysis of PD-1^+^TIM3^+^ terminally exhausted CD8^+^ T cells. (F) Quantification of CXCR2^+^ PMN-MDSC infiltration in metastatic nodules. (G) Schematic of in vitro coculture of FFAR2^fl/fl^ or FFAR2^cko^ PMN-MDSCs with tumor cells. (H) Population of CXCR2^+^ PMN-MDSCs after coculture. (I) Arginase-1 (Arg1) and nitric oxide synthase 2 (NOS2) mRNA expression in PMN-MDSCs after coculture. (J) Quantification of l-arginine uptake by PMN-MDSCs using isotope or fluorescent probes. (K) Schematic of secondary coculture of PMN-MDSCs with CD8^+^ T cells in vitro. (L) Carboxyfluorescein diacetate succinimidyl ester (CFSE)-based assessment of CD8^+^ T cell proliferation. (M) Flow cytometry analysis of GZMB^+^TCF7^+^ effector CD8^+^ T cell subset. (N) Lactate dehydrogenase (LDH) release assay measuring CD8^+^ T cell cytotoxicity. (O) Flow cytometry detection of CD3ζ chain expression. (P) Western blot (WB) analysis of p-ZAP70/p-LCK and total protein levels in T cells. (Q) Measurement of intracellular Ca^2+^ influx in T cells using fluorescent calcium indicators. (R) Quantification of extracellular l-arginine levels in tumor interstitial fluid from FFAR2^fl/fl^ control and FFAR2^cko^ mice bearing SETD2-KO tumors. (S) Representative multiplex immunofluorescence images showing Ly6G^+^ PMN-MDSC-like cells and CD8^+^ T cells in lung metastatic nodules from FFAR2^fl/fl^ control and FFAR2^cko^ mice bearing SETD2-KO tumors. Data are presented as bars and dots, with each dot representing data from one independent experiment. *P < 0.05, **P < 0.01, ***P < 0.001, and ****P < 0.0001. ns, nonsignificant.

To further validate whether FFAR2 participates in SETD2-driven PMN-MDSC activation via the CXCL1/2/5–CXCR2 axis and subsequent T cell exhaustion, Ly6G^Cre^ mice were crossed with FFAR2^fl/fl^ mice to create Ly6G^+^ granulocytic/PMN-MDSC-like cell-specific FFAR2 conditional KO (FFAR2^cko^) mice. NTC or SETD2-KO 3LL cells (2M) were injected intravenously into FFAR2^cko^ and FFAR2^fl/fl^ (control) mice (Fig. 5A). In FFAR2^cko^ mice, metastatic nodules formed by both NTC and SETD2-KO cells were significantly reduced, with a greater reduction observed for SETD2-KO cells compared with NTC cells (Fig. 5B and C).

Within FFAR2^cko^ nodules, the proportion of GZMB^+^TCF7^+^ effector CD8^+^ T cells was significantly increased, whereas terminally exhausted PD-1^+^TIM3^+^ CD8^+^ T cells were markedly decreased (Fig. 5D and E). However, the abundance of CXCR2^+^ PMN-MDSCs was unchanged between FFAR2^fl/fl^ and FFAR2^cko^ mice (Fig. 5F).

SETD2-OE KLN205 cells were also injected into FFAR2^fl/fl^ or FFAR2^cko^ mice (Fig. S8A). In this setting, metastatic nodules formed by NTC cells were significantly reduced in FFAR2^cko^ mice. However, SETD2-OE KLN205 cells produced comparable numbers of nodules in both mouse genotypes (Fig. S8B and C). This finding likely reflects the relatively low baseline CXCL1/2/5 secretion in SETD2-OE cells, resulting in fewer CXCR2^+^ PMN-MDSCs in their nodules and reduced l-arginine uptake; thus, FFAR2 deletion had only minor impact on CD8^+^ T cell responses. Consistently, the frequencies of GZMB^+^TCF7^+^ effector CD8^+^ T cells and PD-1^+^TIM3^+^ terminally exhausted T cells in SETD2-OE nodules were not significantly altered between FFAR2^fl/fl^ and FFAR2^cko^ mice (Fig. S8D and E).

Next, PMN-MDSCs were isolated from FFAR2^fl/fl^ and FFAR2^cko^ mice and cocultured with NTC or SETD2-KO 3LL cells in vitro (Fig. 5G). Consistent with in vivo results, FFAR2 deletion did not affect CXCR2^+^ polarization of PMN-MDSCs (Fig. 5H) nor Arg1 or NOS2 expression (Fig. 5I) but significantly reduced l-arginine uptake (Fig. 5J). Subsequent coculture of these PMN-MDSCs with CD8^+^ T cells (Fig. 5K) demonstrated a marked attenuation of T cell exhaustion in the FFAR2^cko^ group (Fig. 5L to N), with restored expression of CD3ζ chain, p-ZAP70, and p-LCK in T cells (Fig. 5O and P). Moreover, Intracellular Ca^2+^ influx in T cells was significantly enhanced upon coculturing with FFAR2^cko^ PMN-MDSCs (Fig. 5Q), indicating that reduced l-arginine uptake by PMN-MDSCs restored T cell responsiveness to external signals.

Direct quantification of tumor interstitial fluid showed that SETD2-KO tumors in control mice had reduced extracellular l-arginine levels, whereas FFAR2 deletion in Ly6G^+^ granulocytic/PMN-MDSC-like cells partially restored l-arginine availability in the TME (Fig. 5R).

Having established the genetic requirement for FFAR2 in this immunosuppressive program, we next evaluated whether FFAR2 could be pharmacologically targeted in vivo. WT C57BL/6 mice bearing established SETD2-KO 3LL lung metastases were treated daily with the FFAR2 antagonist GLPG0974 or vehicle control. Consistent with the FFAR2^cko^ model, systemic FFAR2 blockade significantly reduced lung metastatic nodule formation (Fig. S9A to C). Flow cytometry analysis further showed increased GZMB^+^TCF7^+^ effector CD8^+^ T cells and reduced PD-1^+^TIM3^+^ terminally exhausted CD8^+^ T cells in GLPG0974-treated tumors (Fig. S9D and E). These findings suggest that the SETD2–CXCL–FFAR2 axis is pharmacologically targetable in vivo.

To further assess the spatial organization of this immune network, we performed multiplex immunofluorescence on lung metastatic nodules. In FFAR2^fl/fl^ control mice bearing SETD2-KO tumors, Ly6G^+^ granulocytic/PMN-MDSC-like cells were abundant, whereas CD8^+^ T cells were relatively sparse. In contrast, tumors from FFAR2^cko^ mice showed increased CD8^+^ T cell infiltration within tumor lesions, despite the presence of Ly6G^+^ cells (Fig. 5S). These spatial data support the conclusion that FFAR2 deletion attenuates the suppressive function of Ly6G^+^ granulocytic/PMN-MDSC-like cells and permits improved CD8^+^ T cell accumulation in the TME.

### SETD2 restricts CXCL1/2/5 secretion via H3K36me3 modification

After establishing that SETD2 loss drives immune evasion by remodeling the metabolic landscape of the TME, the next question focused on the transcriptional origin: How does SETD2, as a histone methyltransferase, precisely restrict the expression of the CXCL1/2/5 chemokine family at the molecular level? Considering that SETD2 is the primary enzyme responsible for H3K36me3 in mammalian cells, it was hypothesized that SETD2 deficiency and the resulting loss of H3K36me3 may disrupt the repressive chromatin state at the CXCL1/2/5 loci.

**Fig. 6.**
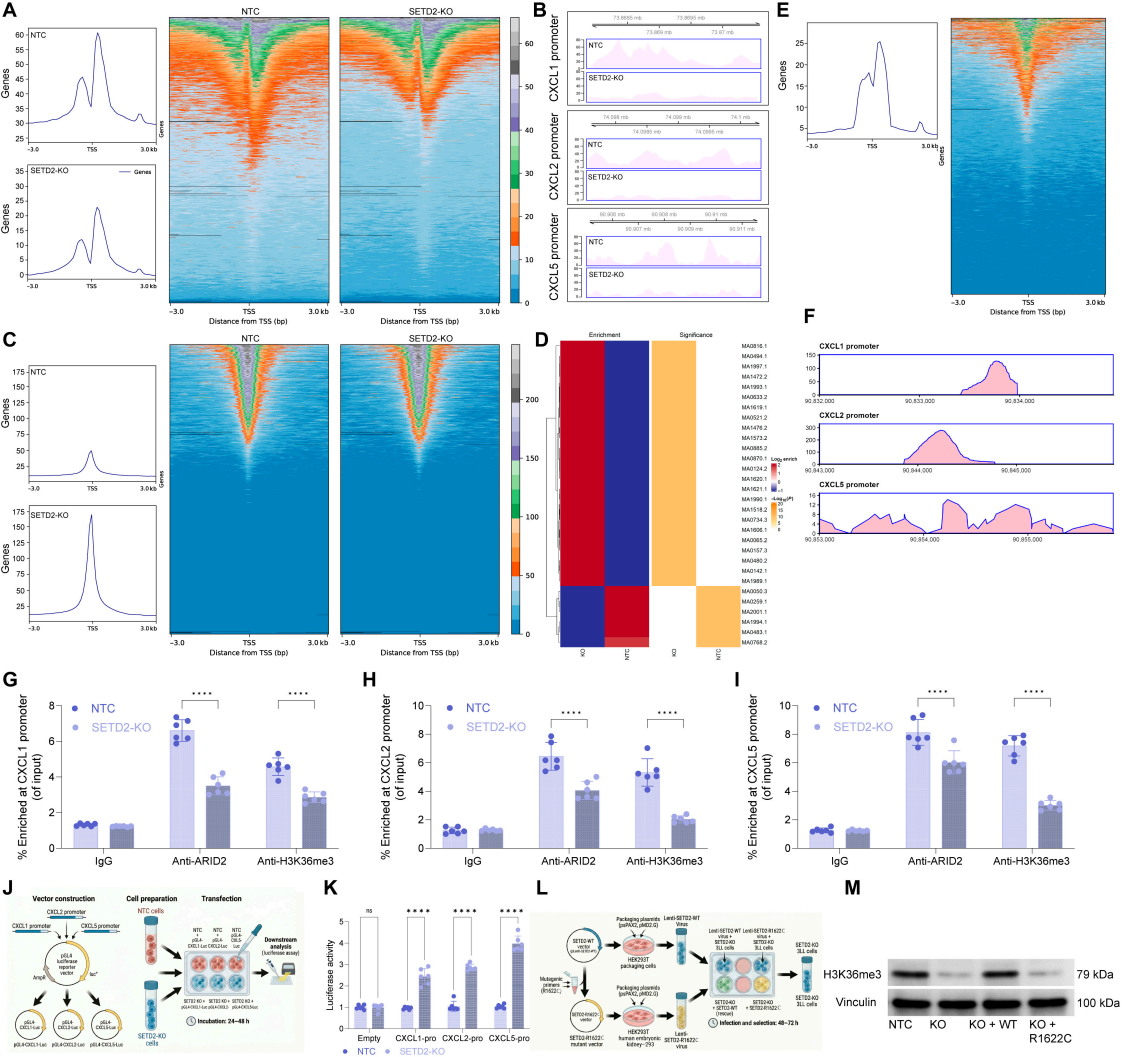
SET domain-containing protein 2 (SETD2) suppresses C-X-C motif chemokine ligand 1/2/5 (CXCL1/2/5) transcription through histone H3 at lysine-36 trimethylation (H3K36me3) deposition. (A) Genome-wide H3K36me3 chromatin immunoprecipitation sequencing (ChIP-seq) profiles in nontargeting control (NTC) and SETD2-knockout (KO) 3LL cells. (B) Tracks showing H3K36me3 enrichment at CXCL1/2/5 loci. (C) Assay for transposase-accessible chromatin with high-throughput sequencing (ATAC-seq) heatmap depicting chromatin accessibility in NTC and SETD2-KO cells. (D) Enrichment tracks of chromatin accessibility at CXCL1/2/5 loci. (E) Genome-wide SETD2 ChIP-seq binding profile at promoter regions. (F) Direct SETD2 binding enrichment at CXCL1/2/5 promoters. (G to I) ChIP-quantitative polymerase chain reaction (qPCR) validation of adenine–thymine-rich interaction domain-containing protein 2 (ARID2) and H3K36me3 enrichment at the Cxcl1, Cxcl2, and Cxcl5 promoters in NTC and SETD2-KO 3LL cells. (J) Schematic of the Cxcl1/2/5 promoter luciferase reporter system. (K) Relative transcriptional activity of Cxcl1, Cxcl2, and Cxcl5 promoters in NTC and SETD2-KO 3LL cells measured by luciferase assay. (L) Experimental design for rescue with wild-type (WT) SETD2 or the SETD2 catalytic domain mutant R1625C. HEK293T, human embryonic kidney 293T. (M) Western blot (WB) analysis of H3K36me3 levels in rescued SETD2-KO 3LL cells. Data are presented as bars and dots, with each dot representing data from one independent experiment. ****P < 0.0001. ns, nonsignificant.

To investigate this, ChIP-seq using anti-H3K36me3 was performed in NTC and SETD2-KO 3LL cells. As expected, SETD2-KO cells exhibited almost complete loss of H3K36me3 across the genome (Fig. 6A and B). Assay for transposase-accessible chromatin with high-throughput sequencing (ATAC-seq) analysis further demonstrated a marked increase in chromatin accessibility at these loci in SETD2-KO cells (Fig. 6C and D), supporting the notion that SETD2 restricts CXCL1/2/5 levels via H3K36me3.

To confirm direct regulation, ChIP-seq with anti-SETD2 demonstrated enrichment of SETD2 at the promoters of CXCL1/2/5 (Fig. 6E and F). Moreover, pharmacologic inhibition of SETD2 using the specific inhibitor EZM0414 in 3LL or KLN205 cells increased CXCL1/2/5 expression and concurrently reduced H3K36me3 levels (Fig. S10A to C) dose dependently. To validate the sequencing results at specific loci, we performed ChIP-qPCR targeting the identified peaks within the Cxcl1, Cxcl2, and Cxcl5 promoters. Consistent with the ChIP-seq profiles, ChIP-qPCR confirmed enrichment of adenine–thymine-rich interaction domain-containing protein 2 (ARID2) (predicted binding partner of SETD2) and H3K36me3 at these loci in NTC cells. In SETD2-KO cells, enrichment of H3K36me3 and ARID2 at the Cxcl1/2/5 promoters was markedly reduced, supporting a requirement for SETD2 in maintaining H3K36me3-associated chromatin regulation at these chemokine loci (Fig. 6G to I).

A CXCL1/2/5 promoter luciferase reporter system was then constructed and transfected into NTC or SETD2-KO cells (Fig. 6J). Luciferase activity was significantly elevated in SETD2-KO cells, indicating derepression of CXCL1/2/5 transcription (Fig. 6K). Previous structure–function analysis has shown that mutation of this conserved SET domain residue, particularly R1625C, impairs SETD2-mediated H3K36me3 and can result in complete loss of H3K36me3 [27]. Therefore, SETD2 R1625C catalytic mutant or WT SETD2 constructs were introduced into SETD2-KO 3LL cells (Fig. 6L). Only WT SETD2 restored H3K36me3 levels, whereas the R1625C mutant failed to do so (Fig. 6M). These results conclusively demonstrate that SETD2 relies on its histone methyltransferase activity to deposit H3K36me3 at CXCL1/2/5 promoters, thereby restricting transcriptional initiation.

### SETD2 recruits ARID2 to compact chromatin and restrict CXCL1/2/5 transcription

Given that the R1625C site of SETD2 resides within the SET domain, a region typically involved in polymerase II binding, and noting from Fig. 6 that SETD2-KO cells show markedly increased chromatin accessibility, it indicates that SETD2 does not act alone. H3K36me3 is usually associated with transcriptional elongation, and its “repressive” function in lung cancer likely depends on a more complex network of protein interactions.

**Fig. 7.**
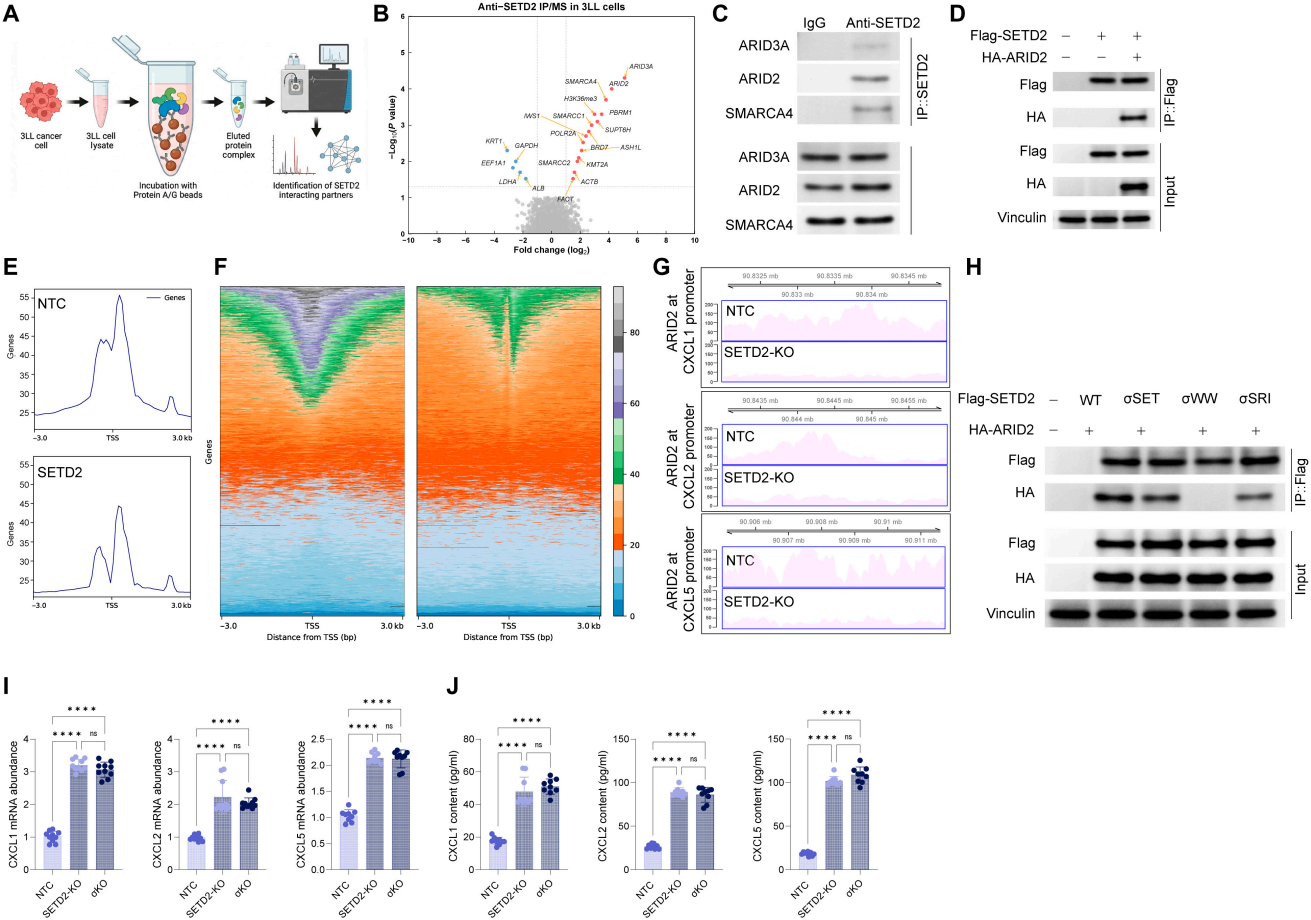
SET domain-containing protein 2 (SETD2) recruits adenine–thymine-rich interaction domain-containing protein 2 (ARID2) via its WW domain to compact chromatin and restrict chemokine transcription. (A) Schematic of anti-SETD2 coimmunoprecipitation (Co-IP) coupled with liquid chromatography-tandem mass spectrometry (LC-MS/MS) in 3LL cells. (B) Top candidate SETD2-interacting proteins identified by mass spectrometry. (C) Endogenous IP validation of physical interaction between SETD2 and ARID2. (D) Exogenous Co-IP in 293T cells overexpressing Flag–SETD2 and hemagglutinin (HA)–ARID2. (E) ARID2 chromatin immunoprecipitation sequencing (ChIP-seq) genome-wide enrichment profile. (F) Heatmap comparing ARID2 binding in nontargeting control (NTC) versus SETD2-knockout (KO) cells. (G) Tracks showing ARID2 binding at C-X-C motif chemokine ligand 1/2/5 (CXCL1/2/5) promoters. (H) Flag-IP analysis of SETD2 deletion mutants (dSET, dWW, and dSRI) for ARID2 binding. (I) Quantitative polymerase chain reaction (qPCR) quantification of CXCL1/2/5 expression in NTC versus SETD2-KO/ARID2–SETD2-double knockout (dKO) cells. (J) Enzyme-linked immunosorbent assay (ELISA) measurement of CXCL1/2/5 protein concentrations in SETD2/ARID2 dKO cells. Data are presented as bars and dots, with each dot representing data from one independent experiment. ****P < 0.0001. ns, nonsignificant.

We hypothesized that SETD2 uses H3K36me3 as a “molecular anchor” to recruit chromatin remodelers, thereby establishing a closed, repressive epigenetic barrier. To test this, coimmunoprecipitation (Co-IP) using anti-SETD2 followed by liquid chromatography-mass spectrometry (LC-MS) was performed in 3LL cells (Fig. 7A), identifying ARID2, SMARCA4 (SWI/SNF-related, matrix-associated, actin-dependent regulator of chromatin, subfamily A, member 4), and ARID3A as the top 3 interacting candidates (Fig. 7B). Further endogenous IP using anti-SETD2 confirmed a robust physical interaction between endogenous SETD2 and ARID2 (Fig. 7C).

Analysis of 2 anti-PD-1 therapy cohorts suggested that nonresponders had significantly lower ARID2 expression than responders (Fig. S11A and B), and stable disease, partial response, and complete response patients exhibited progressively higher ARID2 levels (Fig. S11C and D). ARID2 expression was also positively correlated with cytotoxic T lymphocyte infiltration (Fig. S11E).

Exogenous coexpression of Flag–SETD2 and hemagglutinin (HA)–ARID2 in 293T cells confirmed physical interaction between SETD2 and ARID2 (Fig. 7D). ChIP-seq using anti-ARID2 revealed that in SETD2-KO cells, ARID2 genome-wide binding was substantially reduced (Fig. 7E and F), including at CXCL1/2/5 promoters (Fig. 7G). Furthermore, ARID2 KO in 3LL cells led to increased CXCL1/2/5 expression (Fig. S11F and G), whereas ARID2 overexpression in KLN205 cells decreased CXCL1/2/5 expression (Fig. S11H and I). These data support the model that SETD2 recruits ARID2 to repress CXCL1/2/5 transcription.

To determine whether ARID2 recruitment depends on SETD2-mediated H3K36me3 deposition or on protein association with SETD2, we generated SETD2 deletion mutants lacking the SET, WW, or SRI domain. Co-IP analysis showed that deletion of the SET catalytic domain did not abolish ARID2 association with SETD2, whereas deletion of the WW domain markedly disrupted this interaction. These results suggest that ARID2 is recruited through a SETD2 WW-domain-dependent association, rather than through direct recognition of the H3K36me3 mark catalyzed by SETD2 (Fig. 7H).

To genetically test epistasis, ARID2 was knocked out in SETD2-KO 3LL cells (double knockout [dKO] cells). CXCL1/2/5 expression in dKO cells was comparable to SETD2-KO cells alone (Fig. 7I and J), demonstrating a nonadditive effect and confirming that ARID2 is a core downstream effector of SETD2 in restricting CXCL1/2/5 transcription.

To examine the in vivo relevance, NTC, ARID2 KO, and dKO 3LL cells were injected into C57BL/6 mice (Fig. S12A). Both ARID2 KO and dKO cells formed significantly more lung nodules (Fig. S12B and C), with increased infiltration of CXCR2^+^ PMN-MDSCs (Fig. S12D) and decreased numbers of GZMB^+^TCF7^+^ CD8^+^ effector T cells (Fig. S12E).

To further validate the clinical relevance of the SETD2–CXCL–FFAR2 axis, we performed spatial multiplexed immunofluorescence (mIF) on human non-small-cell lung carcinoma (NSCLC) tissue microarrays. Tumors with high SETD2 expression tended to show lower FFAR2 and CXCR2 fluorescence intensity, whereas SETD2-low tumors showed higher FFAR2 and CXCR2 signals. SETD2 expression was also positively associated with CD8^+^ T cell infiltration (Fig. S13A to D).

## Discussion

The systematic identification of mechanisms that govern immune evasion is important for understanding and potentially overcoming therapeutic resistance in lung cancer. In this paper, we utilized an in vivo CRISPR-negative selection screen to identify SETD2 as an important epigenetic regulator whose loss facilitates lung cancer metastasis by shaping an immunosuppressive microenvironment. Our findings delineate a multistep cascade: SETD2 loss reduces H3K36me3-associated chromatin regulation at the Cxcl1/2/5 loci, increases CXCL1/2/5 expression, promotes recruitment of Ly6G^+^ granulocytic/PMN-MDSC-like cells, and enhances FFAR2-associated arginine uptake in these cells, thereby limiting extracellular l-arginine availability and impairing CD8^+^ T cell function.

Our initial observation that low-dose 3LL cells only colonize the lungs of T-cell-deficient mice underscores the immune-mediated barrier that circulating tumor cells must overcome to establish metastatic foci. Through our mEpi driver library screen, SETD2 emerged as a candidate gene preferentially enriched in metastatic lesions from immune-intact mice. This suggests that while SETD2 loss may provide some intrinsic growth advantages, its most critical role in the context of metastasis is promoting adaptation to T-cell-mediated immune pressure. This aligns with previous and recent genomic studies identifying SETD2 as a frequently mutated tumor inhibitor in lung cancer [28–30]. Notably, we observed that SETD2 loss promotes metastasis in immune-intact mice but not in T-cell-depleted mice, whereas SETD2 overexpression suppressed metastasis in immune-intact mice. These findings suggest that SETD2 is not only involved in genomic or chromatin stability but also contributes to regulation of the TME. The clinical relevance of this pathway is supported by the analysis of anti-PD-1 cohorts, in which lower SETD2 expression was correlated with poorer response to therapy [31]. Nevertheless, SETD2 status should be considered a candidate immunotherapy-associated biomarker that requires further validation in larger treatment-annotated cohorts.

Continuous antigen exposure and persistent inflammation within the TME can cause the exhaustion of CD8^+^ T cells, converting them into dysfunctional exhausted T cells [32,33]. These exhausted cells show increased levels of inhibitory receptors, decreased self-renewal ability, and a progressive decline in effector functions, making them less capable of combating tumors [33]. Exhausted CD8^+^ T cells are typically categorized into 2 types: progenitor exhausted (TIM3^−^) and terminally exhausted (TIM3^+^), each exhibiting unique characteristics [34]. Progenitor exhausted cells retain some responsiveness to immune checkpoint inhibitors, which can help control tumor growth, while terminally exhausted cells, with diminished proliferative potential, are less responsive to most forms of immunotherapy [35–38]. We identified that tumor nodules derived from SETD2-KO cells presented increased populations of both progenitor and terminally exhausted CD8^+^ T cells; however, coculturing with SETD2-KO 3LL cells did not significantly affect T cell activity in vitro, indicating that the T cell dysfunction induced by SETD2 loss was not mediated by direct tumor cell–T cell interactions.

Bulk RNA-seq and CellChat analyses identified up-regulated CXCL1/2/5 signaling in SETD2-KO tumors. These chemokines recruit Ly6G^+^ granulocytic myeloid cells, including PMN-MDSCs, via CXCR2. PMN-MDSCs are a subset of MDSCs that are up-regulated in patients with cancer, playing a key role in immune suppression [39]. These cells, which have multilobed nuclei similar to neutrophils [40], inhibit T cell function and help form an immunosuppressive TME by secreting suppressive cytokines, promoting T cell apoptosis, and enhancing the abundance of immune checkpoint proteins [41,42]. The interaction between tumor cells and neutrophil-lineage cells is increasingly recognized as an important driver of tumor progression; for example, tumor-derived CXCL1/8 can recruit tumor-associated neutrophils and promote lymphangiogenesis and lymphatic metastasis in bladder cancer [43]. CXCR2, a G-protein-coupled receptor, is implicated in the activity of various human C-X-C chemokines [44]. The CXCR2 pathway, along with its ligands, exerts a crucial function in the chemotaxis of neutrophils and MDSCs, contributing to tumor angiogenesis, progression, and tolerance to chemotherapy [45,46].

The consequence of this CXCL1/2/5 up-regulation is increased recruitment of CXCR2^+^ Ly6G^+^ granulocytic/PMN-MDSC-like cells. We identified increased CXCR2^+^ PMN-MDSCs in both coculture systems with SETD2-KO 3LL cells in vitro and in SETD2-KO cell-derived metastatic nodules in vivo. This population of cells also showed a stronger suppressive effect on CD8^+^ T cell proliferation and function, consistent with previous reports [47,48]. Moreover, while MDSCs are known inhabitants of the lung TME, our work identifies a unique metabolic reprogramming within these cells upon recruitment. We observed that the SETD2-deficient TME becomes depleted of extracellular l-arginine. Recruited PMN-MDSCs expressed high levels of Arg1/NOS2 and showed increased l-arginine uptake, leading to the formation of an arginine-depleted TME. It is well recognized that CD8^+^ T cells are hypersensitive to extracellular l-arginine levels [49]. Arginine is essential for the stabilization of the CD3ζ chain [26], the subsequent phosphorylation of ZAP70 and LCK, and T cell function. By depleting extracellular arginine, SETD2-deficient tumors effectively “unplug” the T cell’s signaling machinery before it can even engage the tumor cell. Our CellChat analysis confirmed this, showing that PMN-MDSCs transmit dominant inhibitory signals to T cells, culminating in terminal exhaustion.

Another novel finding of the study is the identification of FFAR2 as a critical downstream effector in PMN-MDSCs. FFAR2, also known as G-protein-coupled receptor 43 (GPR43), is a short-chain fatty-acid-activated G-protein-coupled receptor (GPCR) expressed in immune and metabolic tissues rather than a canonical amino acid transporter [50]. FFAR2/GPR43 has been reported to regulate immune and metabolic signaling through GPCR-dependent pathways, including cyclic adenosine monophosphate, mitogen-activated protein kinase, and Ca^2+^-associated signaling [51,52]. In our study, FFAR2 was up-regulated in CXCR2^+^ PMN-MDSC-like cells. Conditional FFAR2 deletion in Ly6G^+^ granulocytic cells did not reduce the abundance of CXCR2^+^ PMN-MDSC cells or Arg1/Nos2 expression, but it reduced l-arginine uptake and restored CD8^+^ T cell function. In addition, acute GLPG0974 treatment markedly reduced l-arginine uptake in CXCR2^+^ PMN-MDSC cells. Because this short-term inhibition is unlikely to reflect transcriptional remodeling, these data suggest that FFAR2 rapidly regulates arginine uptake-associated metabolic activity. However, they do not establish FFAR2 as a direct physical arginine transporter. A more plausible interpretation is that FFAR2-dependent GPCR signaling modulates canonical cationic amino acid transport pathways, transporter trafficking, membrane potential, or other rapid metabolic processes. Cationic amino acid transporters, including cationic amino acid transporter family transporters encoded by solute carrier family 7 (SLC7) genes, are established mediators of arginine transport [53,54]. Thus, FFAR2 appears to act as a metabolic signaling regulator that promotes the arginine-consumptive phenotype of PMN-MDSC cells. Further studies examining solute carrier family 7/cationic amino acid transporter transporter expression, transporter activity, and flux competition will be required to define the precise mechanism.

Regarding the role of SETD2 in regulating CXCL1/2/5 chemokines, we identified that SETD2 contributes to H3K36me3 deposition and chromatin regulation at these loci. Although H3K36me3 is commonly enriched across actively transcribed gene bodies, SETD2-dependent H3K36me3 can also contribute to transcriptional fidelity, suppression of cryptic intragenic transcription, and restoration of a less permissive chromatin state after RNA polymerase II passage [17,55–57]. In our model, SETD2 loss reduced H3K36me3 occupancy and increased chromatin accessibility at the Cxcl1/2/5 loci, supporting a context-dependent role for SETD2 in restraining chemokine transcription. Moreover, our data suggest that SETD2 acts as a chromatin-associated scaffold by associating with the polybromo-associated BAF complex complex component ARID2 through its WW domain. SETD2–ARID2 association may further contribute to reduced chromatin accessibility at Cxcl1/2/5 regulatory regions.

ARID2 has been implicated in lung cancer biology, and ARID2 loss has been reported to promote malignant progression in lung adenocarcinoma models (PubMed identifier: 34858604). However, the prognostic significance of ARID2 alterations in human lung cancer remains incompletely defined. Our in vivo epistasis experiments support a mechanistic role for ARID2 downstream of SETD2 in limiting CXCL1/2/5 expression, PMN-MDSC recruitment, and metastatic progression. In this scenario, H3K36me3 deposition provides the SETD2-dependent chromatin context required to restrain CXCL1/2/5 transcription, while ARID2 association provides an additional chromatin-remodeling mechanism that limits chromatin accessibility at these loci. To extend these findings to human disease, we performed spatial mIF analysis using human NSCLC tissue microarrays. Consistent with our experimental models, SETD2-low tumors showed higher FFAR2 and CXCR2 signals, whereas SETD2-high tumors showed lower FFAR2 and CXCR2 fluorescence intensity. SETD2 expression was also positively associated with CD8^+^ T cell infiltration. These findings provide human tissue-level support for the proposed SETD2–CXCL–CXCR2–FFAR2 axis and suggest that reduced SETD2 expression may be linked to enhanced granulocytic myeloid/FFAR2-associated signaling and impaired CD8^+^ T cell infiltration in NSCLC.

Several limitations should be noted. Ly6G-based antibody depletion and Ly6G-Cre-mediated conditional deletion are not exclusively specific for PMN-MDSCs and may also affect broader neutrophil-lineage cells. We therefore interpreted these experiments as targeting Ly6G^+^ granulocytic myeloid cells, including PMN-MDSC-like populations, and used flow cytometry to define the tumor-infiltrating CD45^+^Ly6G^+^Ly6C^−^Gr1^+^CD11b^+^ population. Moreover, we did not include a formal prognostic model or immunotherapy-response prediction using public cohorts. A reliable clinical prediction model would require large, independent, treatment-annotated cohorts with harmonized survival and anti-PD-1 response data, as well as accurate assessment of PMN-MDSC abundance and metabolic activity. We would like to fill these gaps in future investigation endeavors.

In summary, this study highlights SETD2 as a critical epigenetic regulator that limits lung cancer metastasis by maintaining a TME permissive to CD8^+^ T cell activity. We reveal an epigenetic–metabolic regulatory axis in which SETD2 loss increases CXCL1/2/5 expression and promotes recruitment of PMN-MDSCs. These cells, influenced by CXCR2–STAT3–FFAR2-associated signaling, increase l-arginine uptake and reduce extracellular arginine availability, leading to impaired CD3ζ-associated proximal T cell receptor/CD3 signaling and CD8^+^ T cell dysfunction. Clinically, SETD2 and ARID2 alterations, CXCL1/2/5 expression, FFAR2 activity, and PMN-MDSC signatures warrant further evaluation as candidate biomarkers or therapeutic vulnerabilities. Our genetic and pharmacological data suggest that FFAR2 inhibition may help restore arginine availability and improve CD8^+^ T cell function in SETD2-deficient tumors, but its therapeutic value will require further validation in clinically relevant models and treatment-annotated patient cohorts.

## Materials and Methods

### Cell line culture and quality control

The murine Lewis lung carcinoma cell line 3LL (VCM00009; C57BL/6 background), KLN205 (VCM00096; DBA/2 background), and human embryonic kidney cells 293T (VCH00001) used in this study were purchased from ViCell Biotechnologies, Henan, China. The 3LL and 293T cells were maintained in high-glucose Dulbecco’s modified Eagle’s medium (#11965092, Gibco, Thermo Fisher Scientific) enriched with 10% fetal bovine serum (FBS; Gibco, Thermo Fisher Scientific) and 1% antibiotics (penicillin–streptomycin; Gibco). The KLN205 cells were cultured in minimum essential medium containing 10% FBS. All cell lines were kept in a humidified 37 °C incubator supplied with 5% CO_2_ (Sanyo, Osaka, Japan). Authentication of the cell lines was performed via short tandem repeat profiling, and regular testing confirmed the absence of mycoplasma contamination. Cell passages were kept within 15 generations, and all experiments were conducted during the exponentially growing phase.

### Lentiviral construction, packaging, and stable cell line selection

sgRNAs targeting SETD2 (5′-GGAGACCAAGGTGGAGTGTC-3′) and ARID2 (5′-GTAGCCTCCAGCATGTAGAA-3′) were cloned into BsmBI-linearized lentiCRISPR v2 (#52961, Addgene) using T4 DNA Ligase (#M0202, New England Biolabs [NEB]). Overexpression vectors and mutants were constructed in pLenti-EF1α-MCS-IRES-Puro backbone and sequence-verified. Lentivirus was packaged in 293T cells using Lipofectamine 3000 (#L3000015, Invitrogen): 10 μg of transfer plasmid + 7.5 μg of psPAX2 (#12260, Addgene) + 2.5 μg of pMD2.G (#12259, Addgene), diluted in 1 ml of Opti-MEM (#31985070, Gibco). Target cells (3LL and KLN205; 2 × 10^5^ cells per well in 6-well plates) were infected with viral supernatant containing polybrene (#TR-1003-G, Sigma-Aldrich; 8 μg/ml). Medium was replaced after 24 h. Puromycin (#P8833, Sigma-Aldrich; 2 μg/ml) or blasticidin (#A1113903, Invitrogen; 5 μg/ml) selection began 48 h postinfection and continued for 10 to 14 d to generate stable lines. KO and overexpression were validated by WB using antibodies against SETD2 (ab69649, Abcam), H3K36me3 (#4909, Cell Signaling Technology [CST]), and ARID2 (sc-166117, Santa Cruz Biotechnology).

### Experimental animals and lung metastasis assay

All animal experiments were ratified by the Institutional Animal Care and Use Committee. Male C57BL/6J and BALB/c nude mice (6 to 8 weeks) were provided by Vital River Laboratory Animal Technology (Beijing, China). A Ly6G^+^ granulocytic myeloid cell-specific FFAR2^cko^ mouse model (Ly6G-Cre; FFAR2^fl/fl^), encompassing PMN-MDSC populations, was generated by crossing Ly6G-Cre transgenic mice with FFAR2^fl/fl^ mice (the Jackson Laboratory) and genotyped by PCR.

Mice were maintained under specific-pathogen-free conditions at 22 ± 2 °C, 50 ± 10% humidity, and 12-h light/dark cycle, with drinking water and pellet feed ad libitum. Log-phase 3LL or KLN205 cells were digested with 0.25% trypsin-EDTA (Gibco), neutralized with 10% FBS medium, centrifuged (300*g* for 5 min at 4 °C), washed, and resuspended in phosphate-buffered saline (PBS). Viability was >95% by Trypan Blue (Gibco). Inoculation densities were as follows: 3LL gradient groups, 1 × 10^6^, 2 × 10^6^, and 4 × 10^6^; standard, 2 × 10^6^; KLN205, 2 × 10^6^. Tail veins were warmed with an infrared lamp; 200 μl of cell suspension was injected slowly via 27-gauge needle (BD Biosciences). Mice were euthanized by CO_2_ overdose or cervical dislocation 14 to 21 d postinjection (based on tumor burden/health). Lungs were excised, weighed, fixed in Bouin’s solution (#HT10132, Sigma-Aldrich) for 24 h, and surface metastatic nodules (>0.1 mm) were blind counted by 2 researchers under a dissection microscope (Olympus).

To assess the preclinical therapeutic efficacy of systemic FFAR2 inhibition, C57BL/6J mice received tail vein injection of 2 × 10^6^ SETD2-KO 3LL cells and were subjected to daily intraperitoneal injections of the highly selective FFAR2 antagonist GLPG0974 (10 mg/kg; formulated in 5% dimethyl sulfoxide [DMSO], 40% polyethylene glycol, molecular weight 300, 5% Tween 80, and 50% saline) 3 d post-cell-inoculation. The control group received equivalent volumes of the vehicle. Treatments were administered daily until the experimental endpoint (day 21), at which point lungs were harvested for metastatic nodule quantification and flow cytometry analysis.

### In vivo epigenetic CRISPR screening

An mEpi driver library (159 epigenetic genes, 1,684 sgRNAs, ~4 sgRNAs/gene, and 100 NTCs) was used for in vivo negative selection in lung metastasis. Library plasmids were cotransfected with psPAX2 and pMD2.G into 293T cells to produce lentivirus. Log-phase 3LL cells were infected at a multiplicity of infection of <0.3 to achieve single-sgRNA integration, selected with puromycin (2 μg/ml). A “day 0” sample was collected, and library coverage was validated by high-throughput sequencing (>95% sgRNA representation and >100 reads/sgRNA).

Selected 3LL cells (1 × 10^8^) were washed 3 times with PBS and resuspended; 2 × 10^6^ cells were injected via tail vein (27-gauge needle; BD Biosciences) into 6- to 8-week male C57BL/6J WT (immune-intact) or T-cell-depleted mice (pretreated with anti-CD4/CD8-neutralizing antibodies, Bio X Cell). Each group had no less than 6 mice (>100-fold sgRNA coverage). After 14 to 21 d, mice were euthanized; 50 metastatic foci per WT mouse were microdissected. The DNeasy Blood & Tissue Kit (#69504, QIAGEN) was utilized for genomic DNA extraction. Sequencing libraries were prepared by 2-step PCR: (a) sgRNA amplification with custom primers and (b) addition of Illumina P5/P7 adapters/indexes. Libraries were sequenced on Illumina NovaSeq 6000. Data were analyzed with MAGeCK (v0.5.9). Hits required ≥2 independent sgRNAs significantly enriched in metastases, a frequency of >40% in WT mice, and >20% in T-cell-depleted mice.

### In vivo immune cell depletion and neutralization

For in vivo chemokine blockade, mice bearing established SETD2-KO 3LL tumors were treated with a neutralizing antibody cocktail targeting CXCL1, CXCL2, and CXCL5 (R&D Systems; 50 μg each per mouse, administered intraperitoneally every 3 d starting on day 3 postinoculation). Control mice received equivalent doses of isotype-matched immunoglobulin G (IgG). Validation of Ly6G^+^ cellular depletion (clone 1A8) was strictly confirmed via flow cytometry of peripheral blood and tumor digests, ensuring >98% clearance of the CD45^+^CD11b^+^Ly6G^+^ compartment prior to downstream analyses. Neutralizing antibodies were administered intraperitoneally to deplete immune subsets. T cell depletion was performed by administering an initial dose of 200 μg of anti-CD4 (GK1.5) + 200 μg of anti-CD8 (2.43) (Bio X Cell) 48 h before tumor injection, followed by maintenance doses every 3 d postinjection. PMN-MDSC depletion was performed by administering 250 μg of anti-Ly6G (1A8, Bio X Cell) every 3 d, starting day 3 postinoculation. Diluted antibodies were injected (200 μl) via 27-gauge needle (BD Biosciences). Controls received equivalent rat IgG2a/IgG2b isotype (Bio X Cell). Depletion was examined by flow cytometry on peripheral blood (ocular puncture), spleen, and lung metastases: >98% reduction in CD45^+^ CD4^+^ T cells, CD45^+^ CD8^+^ T cells, and CD45^+^ Ly6G^+^ Gr1^+^ PMN-MDSCs required for inclusion in downstream analyses.

### Multicolor flow cytometry

Lung metastasis tissues were mechanically dissected and digested in RPMI 1640 at 37 °C with shaking for 45 min. Cell digests were passed sequentially through 70- and 40-μm cell strainers (Corning) to obtain single-cell suspensions. After centrifugation, red blood cells were lysed by incubating the pellets in ACK lysis buffer (Gibco) for 3 min. The resulting single-cell preparations were then stained with Fixable Viability Dye eFluor 780 (#65-0865-14, eBioscience) on ice, followed by surface antibodies (1:200): anti-CD45 (30-F11), anti-CD8a (53-6.7), anti-PD-1 (29F.1A12), anti-TIM3 (RMT3-23), anti-Ly6G (1A8), anti-Ly6C (HK1.4), anti-CXCR2 (SA044G4), anti-FFAR2 (#BS-13536R, Invitrogen). The Foxp3/Transcription Factor Staining Buffer Set (#00-5523-00, eBioscience) was utilized for intracellular staining (Foxp3, GZMB, and TCF7). Data were read on BD FACSCelesta and analyzed with FlowJo (v10.8).

### Dissociation of tumor-infiltrating leukocytes

Fresh mouse lung metastasis tissues were washed in ice-cold Hanks’ balanced salt solution (HBSS; #14175095, Gibco). Tissues were minced into ~1-mm^3^ fragments with fine surgical scissors in a sterile petri dish (Corning). Minced tissue was loaded into a 50-ml tube containing 5 ml of prewarmed digestion buffer.

Digestion proceeded on an orbital shaker at 37 °C for 35 min at 100*g*. Reaction was stopped with equal volume cold RPMI 1640 + 10% FBS (Gibco). Suspension was passed through 70- and 40-μm cell strainers, centrifuged at 300*g* for 5 min at 4 °C. Pellet was treated with 2 ml of ACK Lysis Buffer (#A1049201, Gibco) for 3 min on ice, diluted with 10 ml of cold PBS. Final pellet was resuspended in PBS + 2% FBS, and viability (>90%) was assessed with Trypan Blue using Countess 3 automated counter (Invitrogen) before flow cytometry or sorting.

### Isolation of primary PMN-MDSCs

Single-cell suspensions from spleen or lung metastases were prepared per MDSC Isolation Kit protocol (#130-094-538, Miltenyi Biotec). Cells (1 × 10^7^) were resuspended in 40 μl of ice-cold MACS Running Buffer [PBS (pH 7.2), 0.5% bovine serum albumin, and 2 mM EDTA; 0.22 μm to filtered through a 0.22-μm filter and degassed]. Anti-Ly6G-Biotin antibodies (10 μl) were added and incubated for 10 min at 4 °C in dark. Anti-Biotin MicroBeads (20 μl) were added and incubated for 15 min at 4 °C. Cells were washed with 10 ml of buffer, centrifuged at 300*g* for 10 min at 4 °C, and resuspended. LS Column (#130-042-401, Miltenyi Biotec) was equilibrated with 3 ml of buffer in QuadroMACS Separator. Cell suspension was applied; column was washed 3× with 3 ml of buffer. Bound cells were eluted with 5 ml of precooled buffer after removing magnet, using plunger. Isolated PMN-MDSCs were resuspended in RPMI 1640 + 10% FBS and kept on ice. Purity (CD45^+^ Ly6G^high^ CD11b^+^) was confirmed to be >96%, and viability remained intact, as determined by flow cytometry (BD FACSCelesta) using anti-CD45 (30-F11), anti-Ly6G (1A8), and anti-CD11b (M1/70) before functional assays.

### Isolation and activation of CD8^+^ T cells

Spleens from healthy C57BL/6 mice were dissociated in ice-cold PBS using syringe plunger on 70-μm strainer (Corning) and washed with RPMI 1640 + 2% FBS. The cell suspension was centrifuged at 300*g* for 5 min; the pellet was treated with 5 ml of ACK Lysis Buffer (#A1049201, Gibco) on ice for 3 min before the reaction was stopped with 10 ml of complete medium. CD8^+^ T cells were isolated using CD8a^+^ T Cell Isolation Kit (#130-104-075, Miltenyi Biotec). Cells were washed, resuspended in 500 μl of buffer, and passed through LS Column. Unlabeled CD8^+^ T cells were collected in flow-through; purity (>95%) was confirmed by flow cytometry with anti-CD8a (53-6.7, BioLegend). Isolated cells were resuspended at 1 × 10^6^ cells/ml in RPMI 1640 + 10% FBS, 1% l-glutamine (Gibco), 1% penicillin–streptomycin, and 50 μM β-mercaptoethanol (#M3148, Sigma-Aldrich). Activation was performed using anti-CD3/CD28 Dynabeads (#11452D, Thermo Fisher Scientific) at 1:1 ratio + recombinant mouse IL-2 (#212-12, PeproTech; 20 ng/ml), followed by incubation at 37 °C for 48 h at 5% CO_2_. Beads were removed with magnetic rack; activated effector CD8^+^ T cells were collected for downstream experiments.

### MDSC–T cell suppression and rescue assay

High-purity CD8^+^ T cells were extracted from the spleens of healthy mice using negative selection with immunomagnetic beads (Miltenyi Biotec). The cells were resuspended in RPMI 1640 and preactivated using anti-CD3/CD28 Dynabeads (#11452D, Thermo Fisher Scientific) in a 1:1 ratio with the beads, and recombinant mouse IL-2 (#212-12, PeproTech) was loaded to a final concentration of 10 ng/ml. The polarized PMN-MDSCs, induced by tumor-cell-conditioned medium or cocultured directly, were then cocultivated with the activated CD8^+^ T cells at a 1:1 or 1:2 effector-to-suppressor ratio in a 96-well U-bottom plate (#3799, Corning, NY, USA) at 37 °C for 72 h at 5% CO_2_.

In rescue experiments investigating arginine metabolic competition, the coculture system was supplemented with either 1 mM of high-purity l-arginine (#A5006, Sigma-Aldrich) or the specific FFAR2 antagonist GLPG0974 (100 nM; Sigma-Aldrich) at the onset of the 72-h cocultivation period. Vehicle controls received equivalent volumes of DMSO. After 72 h, the cells were collected by physical shaking, and T cell proliferation kinetics (carboxyfluorescein diacetate succinimidyl ester [CFSE] dilution) and effector cytokine (GZMB) expression were determined using a BD LSRFortessa flow cytometer. In addition, calcium influx in T cells upon secondary stimulation was monitored in real time using the calcium-sensitive fluorescence probe Fluo-4 acetoxymethyl ester (Invitrogen) to quantitatively evaluate the immunosuppressive polarization degree of PMN-MDSCs and their metabolic deprivation mechanisms.

### Calcium influx kinetics assays

CD8^+^ T cells following coculture were washed twice with prewarmed HBSS containing 1.26 mM CaCl_2_ and 0.49 mM MgCl_2_ (#14025092, Gibco) and resuspended at 2 × 10^6^ cells/ml. Cells were incubated with 2 μM Fluo-4 acetoxymethyl ester for 30 min at 37 °C in the dark, with intermittent mixing. Loading was terminated by adding 5 volumes of HBSS containing 1% FBS, followed by centrifugation (300*g* for 5 min at 4 °C) and 2 washes.

Cells were resuspended in 500 μl of HBSS prior to analysis on a BD LSRFortessa. Baseline fluorescence was recorded for 30 s prior to stimulation with anti-mouse CD3 antibody (clone 145-2C11, #100302, BioLegend) at 5 μg/ml. Fluorescence (fluorescein isothiocyanate [FITC]/Alexa Fluor 488 channel) was continuously recorded for 300 to 600 s to capture peak and plateau phases. Data were processed using FlowJo v10.8 (TreeStar), and calcium flux was quantified by area under the curve and peak fold increase over baseline.

### CFSE proliferation assay

Purified CD8^+^ T cells were rinsed with calcium- and magnesium-free PBS (Gibco), adjusted to a concentration of 1 × 10^7^ cells/ml, and labeled with 5 μM CFSE (#C34554, Invitrogen) for 10 min in the dark. Staining was quenched with cold FBS-containing medium, followed by centrifugation and 3 washes.

Cells were plated at 1 × 10^5^ cells per well in 96-well U-bottom plates (#3799, Corning) and cocultured with tumor-polarized PMN-MDSCs at 1:1 for 72 h. After incubation, cells were stained with Fixable Viability Dye eFluor 780 and analyzed by flow cytometry. Proliferation index and division percentages were calculated on the basis of CFSE dilution using FlowJo v10.8.

### Lactate dehydrogenase release cytotoxicity assay

CD8^+^ T cell cytotoxicity against 3LL tumor cells (NTC or SETD2-KO) was measured via the CytoTox 96 Non-Radioactive Cytotoxicity Assay (#G1780, Promega). Cocultures were set up by combining effector CD8^+^ T cells and target 3LL cells at an E:T ratio of 10:1 in a total volume of 100 μl per well of 96-well V-bottom plates. Control wells included target spontaneous release, target maximum release, effector spontaneous release, and medium background.

After 12 h at 37 °C with 5% CO_2_, plates were centrifuged (250*g* for 5 min), and 50 μl of supernatant was loaded to 96-well flat-bottom plates. The lactate dehydrogenase (LDH) substrate was added, and plates were incubated for 30 min avoiding light exposure, followed by stop solution. Absorbance at 490 nm was read utilizing an EnSpire plate reader (PerkinElmer). Cytotoxicity (%) was calculated according to the manufacturer’s formula based on LDH release.

### l-[^3^H] arginine uptake assay

Competitive arginine uptake by PMN-MDSCs was measured using radioactive isotope tracing. Primary PMN-MDSCs were washed 3 times with prewarmed HBSS (#14025092, Gibco) to induce metabolic starvation and resuspended at 1 × 10^6^ cells per tube.

Cells were incubated with l-[2,3,4,5-^3^H] arginine monohydrochloride (1 μCi/ml) (#NET1123, PerkinElmer; specific activity of >50 Ci/mmol) at 37 °C for 10 min at 5% CO_2_ to capture linear uptake kinetics. Uptake was terminated by adding 1 ml of ice-cold PBS enriched with 10 mM unlabeled l-arginine (Sigma-Aldrich), followed by centrifugation (1,000*g* for 5 min at 4 °C) and 2 washes with cold PBS to remove extracellular and surface-bound tracer.

Cells were lysed in 200 μl of 0.1 M NaOH (Sigma-Aldrich) for 30 min at 23 to 25 °C. Lysates were transferred to scintillation vials containing 3 ml of Ultima Gold scintillation fluid, and radioactivity was quantified utilizing a Tri-Carb 2910 TR liquid scintillation counter. Arginine uptake rates were normalized to total input radioactivity, and total protein content was determined by the Pierce BCA Protein Assay Kit (Thermo Fisher Scientific).

For antagonist validation assays, primary PMN-MDSCs were preincubated with the highly selective FFAR2 antagonist GLPG0974 (100 nM; Sigma-Aldrich) or DMSO vehicle control for 15 min at 37 °C prior to the addition of l-[2,3,4,5-^3^H] arginine.

### Co-IP and LC-MS/MS

To identify SETD2-interacting protein complexes, Co-IP followed by LC-MS/MS was performed. Approximately 1 × 10^7^ exponentially growing 3LL cells or transfected 293T cells were lysed in 1 ml of radioimmunoprecipitation assay buffer for 30 min on ice. Lysates were centrifuged (12,000*g* for 15 min at 4 °C), and protein concentration was analyzed via the Pierce BCA Kit.

Equal amounts of protein (1 mg) were incubated overnight at 4 °C with 5 μg of anti-SETD2 (#ab69649, Abcam) or anti-Flag antibody (#F1804, Sigma-Aldrich). Pierce Protein A/G Magnetic Beads (#88802, Thermo Fisher Scientific; 50 μl) were supplemented and rotated for 2 h at 4 °C. Beads were rinsed with tris-buffered saline containing 0.05% Tween 20.

Bound proteins were eluted at 95 °C for 10 min, separated by SDS-polyacrylamide gel electrophoresis, and subjected to in-gel digestion (dithiothreitol reduction, iodoacetamide alkylation, and overnight trypsin digestion; #V5111, Promega). Peptides were extracted, desalted, and analyzed via an Orbitrap Fusion Lumos Tribrid Mass Spectrometer coupled to an EASY-Spray high-performance liquid chromatography column.

Data-dependent acquisition was performed with precursor scans (mass/charge ratio, 350 to 1,500) at a 120,000 resolution, followed by higher-energy collisional dissociation fragmentation. Raw data were processed using MaxQuant v1.6.17 against the UniProt database with trypsin specificity (≤2 missed cleavages), fixed carbamidomethylation (Cys), variable oxidation (Met), and false discovery rate (FDR) of <1% at protein and peptide levels. Enriched proteins were identified by comparison with IgG controls, and the top candidates included ARID2 and associated complex members.

### Tumor interstitial fluid extraction and arginine quantification

To directly quantify local metabolite concentrations within the TME, tumor interstitial fluid was extracted from freshly excised lung metastatic nodules. Nodules were briefly rinsed in cold PBS to remove surface blood, blotted dry, and placed on preweighed 70-μm cell strainers resting in 50-ml conical tubes. Tissues were subjected to centrifugation at 100*g* for 10 min at 4 °C to collect the interstitial fluid. The collected tumor interstitial fluid was immediately deproteinized using 10-kDa molecular weight cutoff spin filters (Amicon). l-Arginine concentrations in the flow-through were quantified using a specific colorimetric l-arginine assay kit (#ab241028, Abcam) according to the manufacturer’s protocol. Concentrations were normalized to the original wet weight of the corresponding tumor nodules.

### mIF of tissue sections

Paraffin-embedded lung tissues from FFAR2^fl/fl^ and FFAR2^cko^ mice were sectioned at 4 μm in thickness. Following deparaffinization, rehydration, and heat-induced epitope retrieval in citrate buffer (pH 6.0), sections were blocked with 5% normal goat serum. Tissues were coincubated overnight at 4 °C with primary antibodies targeting Ly6G (1:200; Servicebio) and CD8a (1:100; CST). Sections were subsequently incubated with corresponding fluorophore-conjugated secondary antibodies (Alexa Fluor 488 and Alexa Fluor 594; Invitrogen) for 1 h at room temperature. Nuclei were counterstained with 4′,6-diamidino-2-phenylindole. Multispectral images were acquired using a laser scanning confocal microscope (Zeiss LSM 880 or equivalent).

### WB analysis

Protein lysates were separated by SDS-polyacrylamide gel electrophoresis and loaded to 0.22-μm polyvinylidene difluoride membranes (#ISEQ00010, Millipore). Membranes were blocked with 5% bovine serum albumin (#A7906, Sigma-Aldrich) in tris-buffered saline containing 0.05% Tween 20 for 1 h at 23 to 25 °C and probed overnight at 4 °C with the following primary antibodies: anti-SETD2 (1:1,000; #ab69649, Abcam), anti-H3K36me3 (1:2,000; #4909, CST), anti-p-STAT3 Y705 (1:1,000; #9145, CST), anti-p-STAT3 S727 (1:1,000; #9134, CST), anti-STAT3 (1:1,000; #9139, CST), anti-CD3ζ (1:1,000; #88083, CST), anti-p-ZAP70 (1:1,000; #2704, CST), anti-p-LCK (1:1,000; #2751, CST), anti-ARID2 (1:1,000; #sc-166117, Santa Cruz Biotechnology), anti-FFAR2 (1:500; #NBP3-12190, Novus/Bio-Techne), and anti-vinculin (1:2,000; #13901, CST) as a loading control. After washing, horseradish-peroxidase-conjugated secondary antibodies (CST) were applied for 1 h at 23 to 25 °C. Signals were developed using enhanced chemiluminescence and imaged with the ChemiDoc MP Imaging System (Bio-Rad).

### ATAC-sequencing (ATAC-seq) library preparation

ATAC-seq was used to analyze chromatin accessibility in SETD2-deficient cancer cells. Briefly, 50,000 viable (>90%; Trypan Blue; Gibco) single cells were collected and lysed in 50 μl of cold nuclear lysis buffer for 10 min on ice. Nuclei were pelleted (500*g* for 10 min at 4 °C) and resuspended in 50 μl of transposition mixure.

Transposition was performed for 30 min at 37 °C. DNA was purified utilizing the MinElute PCR Purification Kit (#28004, QIAGEN). Amplification was performed with NEBNext High-Fidelity 2× PCR Master Mix (#M0541, NEB) and indexed primers (72 °C for 5 min; 98 °C for 30 s; and 8 to 12 cycles of 98 °C for 10 s, 63 °C for 30 s, and 72 °C for 1 min). Postamplification cleanup was achieved with AMPure XP beads (#A63881, Beckman Coulter; 1:1 ratio).

Library concentration and size distribution were determined with a Qubit 4 Fluorometer (Thermo Fisher Scientific) and Agilent 2100 Bioanalyzer. Libraries showing nucleosome periodicity were sequenced on an Illumina NovaSeq 6000 (PE150). Bioinformatic processing included quality evaluation of raw reads with FastQC (v0.11.9), adapter trimming and quality filtering via Trim Galore (v0.6.6), alignment to the mm10 mouse reference genome using Bowtie2 (v2.4.2), and peak calling with MACS2 (v2.2.7).

### ChIP-seq

Adhering to the instruction manual of the SimpleChIP Plus Enzymatic DNA IP Kit (#9005, CST), cells were cross-linked with 1% formaldehyde (10 min at 23 to 25 °C) and quenched with 0.125 M glycine. Chromatin was digested with micrococcal nuclease and further sheared utilizing a Bioruptor Pico (Diagenode; 30-s on/30-s off, 5 cycles) to 150- to 500-bp fragments.

Then, 10 μg of chromatin were incubated with antibodies against H3K36me3, SETD2, STAT3, or ARID2 and magnetic beads overnight at 4 °C. After DNA purification, libraries were prepared using the NEBNext Kit and sequenced on the Illumina NovaSeq 6000 platform. Peak calling was carried out utilizing MACS2 (v2.2.7), and genomic tracks were generated with deepTools for visualization.

### Dual-luciferase reporter assay

To evaluate the regulatory effect of SETD2 on CXCL1/2/5 promoter activity, ~2-kb promoter regions upstream of the transcription start site were amplified from mouse 3LL genomic DNA and cloned into the pGL4.10[luc2] vector (#E6651, Promega). Constructs were sequence-verified.

NTC or SETD2-KO 3LL cells were plated in 24-well plates (#3524, Corning) at 1 × 10^5^ cells per well (70% to 80% confluence). Cells were cotransfected with 1 μg of promoter–reporter plasmid and 50 ng of pRL-TK (#E2241, Promega) as internal control. Medium was replaced 6 h later, followed by another 24 h.

Cells were lysed for 15 min at 23 to 25 °C. Luciferase activities were measured using the Dual-Luciferase Reporter Assay System (#E1910, Promega) on an EnSpire Multimode Plate Reader (PerkinElmer; 10-s integration). Firefly luciferase activity was normalized to the corresponding *Renilla* luciferase activity, and the resulting values were expressed as fold change relative to the NTC control group.

To validate direct transcriptional regulation, the WT murine Ffar2 promoter region containing the putative STAT3 binding motif and a corresponding mutant sequence with a scrambled STAT3 binding site were synthesized and cloned into the pGL3-basic luciferase reporter vector (Promega). Cells were cotransfected with the respective pGL3-Ffar2 promoter constructs (WT or mutant) and the pRL-TK *Renilla* luciferase control vector using Lipofectamine 3000 (Invitrogen). Following 48 h of incubation, luciferase activity was quantified using the Dual-Luciferase Reporter Assay System (Promega). Firefly luciferase activity was normalized to *Renilla* luciferase activity for each sample.

### scRNA-seq and communication network analysis

To assess TME remodeling following SETD2 deletion, scRNA-seq was performed on freshly isolated NTC and SETD2-KO lung metastatic nodules. Tumors were dissociated and resuspended. After removing dead cells, cell viability (≥90%) and concentration (700 to 1,200 cells/μl) were confirmed using the Countess 3 Automated Cell Counter (Invitrogen).

Approximately 10^4^ live cells were loaded onto the Chromium Controller. Libraries were prepared, quantified, quality-checked on an Agilent 2100 Bioanalyzer, and sequenced as described above. CellRanger v6.0.0 (10x Genomics) was used to process the raw data against mm10. Analyses were performed using Seurat v4.0.0 (R v4.1.0). Cells with 200 to 6,000 genes and <5% mitochondrial content were kept; doublets were removed via DoubletFinder v2.0.3. LogNormalize (scale factor, 10,000) was applied, top 2,000 variable genes were selected, principal components (PC) analysis was performed, and top 30 principal components were used for Uniform Manifold Approximation and Projection (UMAP) and Louvain clustering.

Cell types were annotated using canonical markers (Cd3e/Cd8a for T cells and Ly6g/Ly6c/Itgam for MDSCs). Intercellular communication was inferred using CellChat v1.1.3 with the CellChatDB.mouse database. Ligand–receptor interaction probabilities were calculated using the computeCommunProb function, and interactions involving <10 cells were filtered. To identify major signaling sources and targets within the TME, network centrality was computed utilizing the netAnalysis_computeCentrality function. This function applies graph theory principles to calculate network metrics (out-degree signal senders and in-degree signal receivers) in a weighted directed network. Based on these centrality metrics, downstream analyses were focused on identifying the most highly weighted outgoing signals from tumor cells and incoming inhibitory signals to T cells. Communication differences were visualized using hierarchical clustering and bubble plots.

### Statistical analysis

Quantitative data were collected from a minimum of 6 independent experiments and are presented as means ± SEM. For standard biological assays, comparisons between 2 groups were performed using 2-tailed unpaired Student’s *t* tests, and comparisons among multiple groups were performed using 1-way or 2-way analysis of variance (ANOVA), followed by Tukey’s post hoc test. For bulk RNA-seq, differential expression analysis was performed using DESeq2, with *P* values adjusted by the Benjamini–Hochberg method to control the FDR. For scRNA-seq, cluster marker identification and differential expression analysis were performed using the Wilcoxon rank-sum test in Seurat, with Benjamini–Hochberg correction for multiple testing. For CellChat analysis, ligand–receptor interaction significance was assessed using permutation testing, with *P* < 0.05 considered significant. For ChIP-seq, peaks were called using MACS2 with an FDR threshold of *q* < 0.05. Unless otherwise specified, statistical significance was defined as *P* < 0.05. Statistical analyses were performed using GraphPad Prism 9.0 and R software version 4.2.1.

## Ethical Approval

This study and included experimental procedures were approved by the institutional animal care and use committee of Shanghai Pulmonary Hospital, School of Medicine, Tongji University (approval number: K25-724). All animal housing and experiments were conducted in strict accordance with the institutional guidelines for the care and use of laboratory animals.

## Data Availability

Research data supporting this publication are available upon request.
