# Supplementary material for: Loss of SETD2-mediated H3K36me3 Drives CD8+ T Cell Exhaustion in Lung Cancer through CXCL-dependent PMN-MDSC Recruitment and Arginine Deprivation
Source: Research (Wash D C). 2026 Aug 5;9:1370. doi: 10.34133/research.1370 (PMC13438026; doi:10.34133/research.1370)
Supplement: Supplementary file 1 [file research.1370.f1.zip › Supplementary materials.docx]

**Fig. S1.** T cell-mediated colonization resistance and evaluation of an epigenetic sgRNA library in a lung cancer metastasis model. (A) Schematic of the 3LL cell injection strategy. Murine Lewis lung carcinoma 3LL cells were injected into C57BL/6 mice (2M, 4M, or 6M) or nude mice (2M or 4M) through tail vein at different cell doses. (B-C) HE staining detection (B) and quantification (C) of metastatic nodules formed by 3LL cells in the lungs of C57BL/6 mice or nude mice. (D) Schematic of CD4 or CD8 T-cell depletion in C57BL/6 mice using neutralizing antibodies. (E-F) Flow cytometry validation of T-cell depletion efficiency in peripheral blood (E) and in the lung (F) of mice. (G) Quantification of metastatic nodules formed after low-dose cell injection in WT or T-cell-depleted mice. (H) Flow cytometry analysis of total CD4^+^ and CD8^+^ T cells in normal lung tissue and metastatic lesions. (I) Flow cytometry analysis of Treg cells (Foxp3⁺) and exhausted CD8⁺ T cells (PD1⁺TIM3⁺) in normal lung tissue and metastatic lesions. (J-K) Cumulative distribution of sgRNA coverage assessed by high-throughput sequencing in library-infected cells. Data are presented as bars and dots, with each dot representing data from one independent experiment. *****p* < 0.0001.

**Fig. S2.** Characterization of SETD2 knockout and overexpression cell lines and correlation analysis in TCGA cohorts. (A-D) Analysis of correlations between SETD2 expression and GZMB, GZMA, CD8A, and PRF1 using TCGA-LUAD data. (E) qPCR analysis of SETD2 transcript levels in 3LL and KLN205 cells. (F) Schematic of sgRNA target design for SETD2. (G) WB analysis confirming loss of SETD2 protein and reduction of H3K36me3 in SETD2-KO 3LL cells. (H) WB analysis validation of SETD2 overexpression in KLN205 cells. (I) Schematic of tumor cell tail-vein injection strategy. (J) In vitro proliferation of NTC and SETD2-KO 3LL cells determined using CCK-8 assays. Cell experiments repeated six times. Data are presented as bars and dots, with each dot representing data from one independent experiment. *****p* < 0.0001.

**Fig. S3**. Association between SETD2 expression and immunotherapy response and CTL infiltration in lung cancer patients. (A-B) Meta-analysis of publicly available anti-PD-1 therapy cohorts comparing SETD2 expression between responders and non-responders. (C) Comparison of SETD2 expression levels among SD, PR, and CR patients based on RECIST criteria. (D-F) Correlation analysis between SETD2 expression and CTL infiltration signatures.

**Fig. S4.** Regulation of the immune landscape in metastatic lesions by SETD2 status and establishment of an in vitro co-culture system. (A-B) Flow cytometric quantification of exhausted T cells (A) and Tregs (B) in SETD2-KO metastatic nodules. (C-D) Flow cytometric quantification of exhausted T cells (C) and Tregs (D) in SETD2-OE metastatic nodules. (E) Schematic of the in vitro T-3LL cell co-culture system. (F) CFSE-based assessment of CD8⁺ T cell proliferation in co-culture. (G) Flow cytometric analysis of GZMB⁺ CD8⁺ T cells in co-culture. (H) LDH release assay to measure T-cell-mediated cytotoxicity against tumor cells. Data are presented as bars and dots, with each dot representing data from one independent experiment. *****p* < 0.0001.

**Fig. S5.** PMN-MDSC depletion negates SETD2 loss-induced lung metastasis and T-cell exhaustion. (A) Experimental design illustrating anti-Ly6G-mediated depletion of PMN-MDSCs in mice received SETD2-KO 3LL cell injection. (B) Representative images of lung tissues from each treatment group. (C) Quantification and comparison of lung metastatic nodules with or without anti-Ly6G treatment. (D) Flow cytometric quantification of effector CD8⁺ T cells (GZMB⁺) in metastatic lesions. (E) Flow cytometric quantification of terminally exhausted CD8⁺ T cells (PD1⁺TIM3⁺) in metastatic lesions. PMN-MDSC-like cells (CD45⁺Ly6G⁺Ly6C⁻Gr1⁺CD11b⁺) were sorted from tumors and co-cultured with mouse CD8^+^ T cells in vitro. (F) CFSE dilution assay measuring CD8⁺ T-cell proliferation in co-culture. (G) Flow cytometric analysis of effector (GZMB⁺TCF7⁺) T cells after co-culture (J) and terminally exhausted (PD1⁺TIM3⁺) (K) CD8⁺ T-cell subsets. (H) Mice bearing SETD2-KO tumors were treated with a neutralizing antibody cocktail against CXCL1, CXCL2, and CXCL5 or isotype control. (I) Flow cytometric quantification of CXCR2^+^ PMN-MDSCs in tumors. (J) Flow cytometric quantification of effector CD8⁺ T cells (GZMB⁺TCF7^+^) in tumors. (K) Flow cytometric quantification of progenitor exhausted CD8⁺ T cells (PD1⁺TCF7⁺) in tumors. Data are presented as bars and dots, with each dot representing data from one independent experiment. ****p* < 0.001, *****p* < 0.0001.

**Fig. S6.** Measurement of arginine uptake and rescue of T-cell activation by L-arginine supplementation. (A) Quantification of L-arginine uptake by CXCR2⁺ versus CXCR2⁻ PMN-MDSCs. (B) Flow cytometric analysis of CD3ζ-chain expression in T cells under co-culture with CXCR2⁺ or CXCR2⁻ PMN-MDSCs. (C) WB analysis of p-ZAP70 and p-LCK levels in T cells after co-culture with PMN-MDSCs across groups. (D) Measurement of intracellular Ca²⁺ influx in T cells using fluorescent calcium indicators. (E) Schematic of the L-arginine supplementation rescue experiment. (F) CFSE-based assessment of T-cell proliferation following L-arginine supplementation. (G) Flow cytometric analysis of GZMB⁺TCF7⁺ CD8⁺ T cells after L-arginine supplementation. (H) LDH release assay measuring restoration of T-cell cytotoxic activity after L-arginine supplementation. Data are presented as bars and dots, with each dot representing data from one independent experiment. ****p* < 0.001, *****p* < 0.0001.

**Fig. S7.** Validation of STAT3 blockade in PMN-MDSC metabolic competition and immunosuppressive function. (A) Schematic of Stattic (STAT3 inhibitor) supplementation to the co-culture system of PMN-MDSCs with activated CD8^+^ T cells. (B) Quantification of arginine uptake rates in PMN-MDSCs following Stattic treatment. (C) qPCR analysis of FFAR2 mRNA expression. (D) Measurement of Arg1 and NOS2 enzymatic activity in PMN-MDSCs after STAT3 inhibition. (E) Flow cytometric analysis of the effect of Stattic on the proportion of CXCR2⁺ PMN-MDSCs. (F) Schematic of co-culture of Stattic-treated PMN-MDSCs with CD8⁺ T cells. (G) CFSE-based analysis of T-cell proliferation in co-culture. (H) Flow cytometric analysis of effector and exhausted T-cell subsets. (I) LDH release assay assessing T-cell cytotoxicity in the co-culture system. Data are presented as bars and dots, with each dot representing data from one independent experiment. *****p* < 0.0001. ns, non-significant.

**Fig. S8.** Effects of FFAR2 deletion in the context of SETD2 overexpression on lung cancer growth and T-cell phenotypes. (A) Experimental design for injection of SETD2-OE KLN205 cells into FFAR2^cko^ or FFAR2^fl/fl^ (control) mice. (B) Representative gross images of lungs. (C) Quantification of lung surface metastatic nodules. (D) Flow cytometric analysis of effector CD8⁺ T cells in metastatic nodules. (E) Flow cytometric analysis of terminally exhausted CD8⁺ T cells. Data are presented as bars and dots, with each dot representing data from one independent experiment. *****p* < 0.0001. ns, non-significant.

**Fig. S9.** Pharmacological inhibition of FFAR2 suppresses SETD2-deficient lung metastasis and restores CD8⁺ T-cell function in vivo. (A) Experimental workflow for pharmacological FFAR2 blockade in vivo. WT C57BL/6 mice bearing established SETD2-KO 3LL lung metastases were treated daily with the FFAR2 antagonist GLPG0974 or vehicle control. (B) Representative gross images of lungs from vehicle- and GLPG0974-treated mice. (C) Quantification of lung surface metastatic nodules in vehicle- and GLPG0974-treated mice. (D) Flow cytometric analysis of GZMB⁺TCF7⁺ effector CD8⁺ T cells in lung metastatic nodules after vehicle or GLPG0974 treatment. (E) Flow cytometric analysis of PD1⁺TIM3⁺ terminally exhausted CD8⁺ T cells in lung metastatic nodules after vehicle or GLPG0974 treatment. Data are presented as bars and dots, with each dot representing one mouse or biological replicate, as indicated. *****p* < 0.0001.

**Fig. S10.** Dose-dependent effects of SETD2 inhibitor on CXCL transcription and histone modification. (A) Experimental scheme for treatment of cells with varying doses of the SETD2 inhibitor EZM0414. (B) qPCR analysis of CXCL1/2/5 mRNA under different inhibitor concentrations. (C) WB analysis of H3K36me3 levels after inhibitor treatment. Data are presented as bars and dots, with each dot representing data from one independent experiment. *****p* < 0.0001.

**Fig. S11.** Clinical relevance, regulatory validation, and SETD2 mutant constructs for ARID2. (A-B) Expression of ARID2 in anti-PD-1 therapy responders vs. non-responders. (C-D) ARID2 expression across SD, PR, and CR patient groups. (E) Correlation of ARID2 expression with CTL infiltration. (F) WB validation of ARID2 protein loss in ARID2-KO 3LL cells. (G) qPCR analysis of CXCL1/2/5 transcription upon ARID2 KO. (H) WB validation of ARID2 protein increase in ARID2-OE KLN205 cells. (I) qPCR analysis of CXCL1/2/5 transcription upon ARID2-OE. (J) Schematic of SETD2 domain deletion mutants (AWS-SET, WW, SRI). ****p* < 0.001, *****p* < 0.0001.

**Fig. S12.** Epistasis validation of ARID2 and SETD2 loss in vivo. (A) Experimental design for NTC, ARID2 KO, or dKO cell injection into mice. (B) Representative lung images post-injection. (C) Quantification of lung surface metastatic nodules. (D) Flow cytometry quantification of CXCR2^+^ PMN-MDSC in nodules. (E) Flow cytometry analysis of effector (GZMB^+^) and terminally exhausted (PD1^+^TIM3^+^) T cells in nodules. *****p* < 0.0001. ns, non-significant.

**Fig. S13.** Spatial mIF analysis of the SETD2–CXCR2–FFAR2 axis in human NSCLC TMA. (A) Representative mIF images of human NSCLC TMA stained for SETD2, CXCR2, FFAR2, CD8, and DAPI. (B-D) Correlation analysis between SETD2 fluorescence intensity and that of CXCR2 (B), FFAR2 (C), and CD8 (D) in human NSCLC specimens.
